# Evaluation of metformin for epilepsy prevention or seizure control in people without evidence of diabetes: retrospective cohort study

**DOI:** 10.1136/jnnp-2025-337861

**Published:** 2026-05-13

**Authors:** Gashirai K Mbizvo, Josephine Mayer, Sneha Mary George, Laura Jayne Bonnett, Glen P Martin, Matthew Sperrin, Pietà Georgina Schofield, William Owen Pickrell, Uazman Alam, Nasir Mirza, Iain Edward Buchan, Gregory Y H Lip, Anthony G Marson

**Affiliations:** 1 Liverpool Interdisciplinary Neuroscience Centre, University of Liverpool, Liverpool, UK; 2 Liverpool Centre for Cardiovascular Science, University of Liverpool, Liverpool John Moores University and Liverpool Heart & Chest Hospital, Liverpool, UK; 3 Pharmacology and Therapeutics, Institute of Systems, Molecular and Integrative Biology, University of Liverpool, Liverpool, UK; 4 The Walton Centre NHS Foundation Trust, Liverpool, UK; 5 Department of Health Data Science, Institute of Population Health, University of Liverpool, Liverpool, UK; 6 Division of Informatics, Imaging and Data Science, Faculty of Biology, Medicine and Health, University of Manchester, Manchester Academic Health Science Centre, Manchester, UK; 7 Department of Public Health, Policy and Systems, Institute of Population Health, University of Liverpool, Liverpool, UK; 8 Swansea University Medical School, Singleton Park, Swansea, UK; 9 Department of Neurology, Morriston Hospital, Swansea Bay University Health Board, Swansea, UK; 10 Diabetes & Endocrinology Research and Pain Research Institute, Institute of Life Course and Medical Sciences, University of Liverpool and Liverpool University Hospital NHS Foundation Trust, Liverpool, UK; 11 Danish Centre for Health Services Research, Department of Clinical Medicine, Aalborg University, Aalborg, Denmark

**Keywords:** EPILEPSY, DIABETES MELLITUS, EPIDEMIOLOGY, ANTICONVULSANTS, SEIZURES

## Abstract

**Background:**

Metformin demonstrates antiepileptogenic (primary prevention) and antiseizure (symptom control) properties in animal models of epilepsy. It is unclear whether these effects translate to humans, particularly outside diabetes, where metformin is increasingly prescribed for weight loss for example. We therefore addressed two questions in people without evidence of diabetes: (1) following an incident brain insult (ischaemic stroke, intracranial haemorrhage, head injury or brain tumour), is metformin associated with reduced risk of first recorded seizure, epilepsy diagnosis or antiseizure medication (ASM) initiation? (2) In established epilepsy treated with ASMs, is metformin add-on associated with reduced risk of potentially seizure-related morbidity (coded seizures, emergency department attendances, hospital admissions, falls, injuries, burns or a composite of these) and all-cause mortality?

**Methods:**

We undertook an international retrospective cohort study of real-world healthcare data in TriNetX. Propensity score matched cohorts compared metformin against licensed weight-management comparators. Cox proportional hazards models estimated HRs with 95% CIs for 10 year outcomes.

**Results:**

16 695 participants were included. Following brain insult, metformin was not associated with reduced risk of first recorded seizure, epilepsy diagnosis or ASM initiation. In established epilepsy, metformin add-on was associated with significantly lower hazards of coded seizures (HR 0.778, CI 0.624 to 0.971), emergency attendance or hospital admission (HR 0.696, CI 0.569 to 0.851), falls/injuries/burns (HR 0.677, CI 0.510 to 0.898) and a composite of these events (HR 0.729, CI 0.626 to 0.848; approximate number needed to treat ~6 at 10 years). Mortality did not differ (HR 1.039, CI 0.594 to 1.816).

**Conclusions:**

Metformin could have repurposed benefits in epilepsy. This warrants further investigation.

**WHAT IS ALREADY KNOWN ON THIS TOPIC**

- Metformin, the most widely used therapy for type 2 diabetes mellitus, has shown potential antiepileptogenic (primary prevention) and antiseizure (symptom control) effects in animal models. In these, it appears to attenuate seizure generation and delay epilepsy onset through activation of adenosine monophosphate-activated protein kinase signalling and inhibition of mechanistic target of rapamycin pathways, both of which are frequently dysregulated in epilepsy. It is unclear whether the effects translate to humans, particularly outside diabetes—where it is increasingly used for weight management.

**WHAT THIS STUDY ADDS**

- Metformin add-on therapy to usual antiseizure medication care in people with epilepsy and no evidence of diabetes seems to be associated with lower net seizure-related outcomes (coded seizures, emergency department attendances, hospital admissions, falls, injuries and burns). No signal is seen for antiepileptogenesis in the current study setup.

**HOW THIS STUDY MIGHT AFFECT RESEARCH, PRACTICE OR POLICY**

- If replicated, these findings could support prioritising metformin as a candidate for repurposing trials in epilepsy, particularly in individuals with comorbid obesity, where weight management is already a clinical consideration.

## Introduction

 Epilepsy is one of the most common neurological disorders worldwide, affecting 70 million people.^1^ Disease modification in epilepsy remains a major challenge and an unmet clinical need. It refers to a process by which therapeutic intervention alters the biological mechanisms driving epileptogenesis. This may involve preventing the onset of epilepsy following a brain insult or in high-risk individuals (antiepileptogenesis). Alternatively, it may involve altering the natural history of established epilepsy by achieving sustained reductions in seizure frequency, severity or epilepsy-related comorbidity either through early treatment or when these benefits persist beyond the period of treatment.^2–4^ Epilepsy surgery can be considered disease-modifying as removal of an epileptogenic lesion eliminates the anatomical and biological substrate driving seizures. Indeed, up to 47% of cases treated with epilepsy surgery achieve complete seizure freedom.^5^ However, this approach is not generalisable—only 10%–20% of individuals with epilepsy are eligible for epilepsy surgery.^6 7^ Mechanistic target of rapamycin (mTOR) inhibitors are disease-modifying when epilepsy is associated with tuberous sclerosis complex (TSC), a rare cause of epilepsy affecting 1 in 10 000 individuals (<1% of epilepsy cases).^8^ TSC is caused by pathogenic variants in TSC1 or TSC2, leading to constitutive activation of the mTORC1 pathway. This drives cortical tuber formation, abnormal neuronal development and early-onset, often refractory epilepsy.^8^ mTOR inhibitors, such as everolimus and sirolimus, therefore target the underlying disease biology rather than providing symptomatic seizure suppression. However, their clinical utility is limited by poor tolerability and a substantial adverse effect burden,^9^ and their applicability is confined to a rare monogenic epilepsy, rendering this disease-modifying strategy non-generalisable to the wider epilepsy population.

Metformin, the most widely used treatment for type 2 diabetes mellitus (T2DM), has shown antiepileptogenic (primary prevention) and antiseizure (symptom control) effects in animal models, including rodents and zebrafish.^10^ These suggest metformin attenuates seizure generation, delays epilepsy onset and preserves cognitive function through activation of adenosine monophosphate-activated protein kinase (AMPK) signalling and inhibition of mTOR pathways, which are frequently dysregulated in epilepsy.^11–13^ The activation of AMPK by metformin not only suppresses mTOR, but also engages pathways such as AMPK-PI3K-c-Jun and PGC-1α, which collectively regulate neural excitability, ion channel function and neuroprotection.^10 13^ These mechanisms are critical in modulating epileptogenesis and reducing seizure activity.^10 13^ Metformin also enhances the expression of brain-derived neurotrophic factor, contributing to its neuroprotective effects.^12^ Some of metformin’s therapeutic effect may also be mediated through its inhibition of *ETFDH*, a gene involved in the development of some types of epilepsy.^14^ Despite promising findings in animal models, metformin largely remains untested in human epilepsy.^11 12 15–17^

Although metformin is licensed for the treatment of T2DM and polycystic ovarian syndrome (PCOS), it is increasingly prescribed off-label for weight management in people without diabetes,^18^ particularly in the USA, reflecting its favourable tolerability and long-established safety profile.^19^ Metformin is also used off-label in other non-diabetic metabolic conditions, including non-alcoholic fatty liver disease and metabolic syndrome.^20–22^ This broader metabolic use raises the question of whether metformin could reasonably be evaluated for potential antiepileptogenic or antiseizure effects outside the context of diabetes. Obesity represents a particularly generalisable target in this regard. People with epilepsy have a consistently higher prevalence of obesity compared with the general population, concordant across multiple recent meta-analyses, large cross-sectional studies and Mendelian randomisation analyses, with reported ORs ranging from approximately 1.28 to 2.1 relative to healthy controls.^23–29^ The investigation of drugs beyond their original indication is not without precedent in epilepsy. A small randomised controlled trial in patients with TSC, a condition distinct from diabetes, demonstrated improvements in seizure control with metformin.^15^ Although TSC is rare and cannot be used to support generalisability to the wider epilepsy population, this provides proof of principle that metformin could influence seizure outcomes in a non-diabetic context. However, further studies are still required, particularly as seizure outcomes were not improved in two small studies of Lafora disease (a 12-patient case series and a separate 18-patient cohort).^15 17^ More recently, a randomised trial of 500 mg two times per day metformin in active epilepsy (n=60) in India showed no significant reduction in seizure frequency versus placebo at 6 months (5% vs 0%, p = 0.687), although seizure frequency declined significantly from baseline in the metformin arm.^30^ The authors concluded that larger studies with longer treatment duration and higher doses are warranted. The successful repurposing of metabolic and weight-related therapies in epilepsy has some translational precedent in epilepsy. Fenfluramine was originally developed and marketed for weight loss and was later observed to have antiseizure effects in clinical practice,^31 32^ prompting formal evaluation in epilepsy trials and,^33–35^ ultimately, regulatory approval for treatment of epilepsy associated with both Dravet syndrome and Lennox–Gastaut syndrome.^36 37^

Given the lack of generalisable human evidence for metformin in epilepsy, we sought to address the two key research questions listed below in people without evidence of diabetes. As metformin is increasingly prescribed off-label for weight management, we assumed this to be a common indication for treatment in this non-diabetic population. We therefore used licensed weight-management therapies as active comparators: tirzepatide, liraglutide and semaglutide (GLP-1 receptor agonists that are also licensed treatments for T2DM), orlistat (a lipase inhibitor) and bariatric surgery.^38^

1. **Antiepileptogenesis:** Among people experiencing an incident brain insult (ischaemic stroke, intracranial haemorrhage, head injury or brain tumour), is commencing metformin (vs active weight-management comparators) associated with reduced risk of a first recorded seizure, epilepsy diagnosis or antiseizure medication (ASM) initiation?
2. **Seizure control:** among people with established epilepsy treated with ASMs, is commencing metformin add-on therapy (vs active weight-management comparators) associated with reduced risk of potentially seizure-related morbidities (coded seizures, emergency department attendances, hospital admissions, falls, injuries, burns or a composite of these events) and all-cause mortality? These were the same potentially seizure-related morbidity case definitions we have used in our prior work.^39^

## Methods

### Study design and setting

We undertook a retrospective cohort study of real-world electronic health data from a global network of healthcare organisations (HCOs) held in the TriNetX LIVE platform.^40–43^ This covers ~250 million patients from >130 HCOs across 19 countries predominantly in North America but also South America, Europe, the Middle East, Africa and Asia Pacific.^40^ There are ~70 billion date- and patient-indexed electronic clinical data points held.^40^ Much of the data come from large academic HCOs, although data from smaller rural hospitals are also imported to better reflect patients from all care backgrounds. Similarly, data from both insured and uninsured patients are captured, covering a variety of healthcare settings including emergency care, inpatients, outpatients and primary care. The data imports occur in real-time, meaning results are contemporaneous with frontline clinical progress. Variables held include demographics, diagnoses (using International Classification of Diseases, Tenth Revision, Clinical Modification (ICD-10-CM) codes), drugs (RxNorm-coded), procedures (including Current Procedural Terminology (CPT) and SNOMED codes), laboratory results, genomics, tumour registration and mortality data.^39–42^

### Search strategy, participants and exposures

We queried the TriNetX Global Collaborative Network in January 2026 to construct the two study cohorts below. The coding strategies used to build each cohort are detailed in online supplemental 1. No age restrictions were applied, although TriNetX automatically excludes patients aged >90 years to preserve anonymity. Participants were followed from the cohort-specific index date until the first occurrence of an outcome event, death, censoring at the last available clinical data point or completion of up to 10 years of follow-up.

#### Cohort 1 (antiepileptogenesis)

People without evidence of diabetes experiencing an incident brain insult (ischaemic stroke, intracranial haemorrhage, head injury or brain tumour) who were commenced on metformin versus active weight-management comparators within 3 years after the insult. The date of the first recorded brain insult code in the TriNetX record was taken as the incident brain insult date, with a record-based lookback using all available prior data to exclude prevalent cases. The initiation of metformin or the active weight-management comparator was taken as the index date (start of follow-up), with a record-based lookback using all available prior data to exclude prior treatment (queried from the earliest available date, defaulting to 1900). The 3-year window was selected as a trade-off between exposure ascertainment (as these agents are not licensed for brain insults, initiation will be largely coincidental and thus few will be initiated contemporaneously) and clinical utility (the risk of developing epilepsy will decrease with increasing time since the insult).

To restrict the cohort to individuals without evidence of diabetes, participants were required to have no diagnostic codes for diabetes, pre-diabetes or abnormal blood glucose at any point in their available health record; no HbA1c ≥5.7% or blood glucose ≥100 mg/dL (which are the thresholds for pre-diabetes);^44^ and no exposure to other antidiabetic medications, including insulin, sulfonylureas, meglitinides, thiazolidinediones, α-glucosidase inhibitors, DPP-4 inhibitors, SGLT2 inhibitors or the remaining GLP-1 receptor agonists (full list in online supplemental 1).

#### Cohort 2 (seizure control)

People with epilepsy and no evidence of diabetes who were treated with ASMs and subsequently commenced on metformin add-on therapy versus active weight-management comparators as add-on. The date of the first recorded seizure or epilepsy code in the TriNetX record was taken as the incident disease date, with a record-based lookback using all available prior data to exclude prevalent cases. The initiation of metformin or the active weight-management comparator was taken as the index date (start of follow-up), with a record-based lookback using all available prior data to exclude prior treatment. No time restriction was placed between the incident disease date and the index treatment date in order to maximise generalisability to real-world clinical scenarios, where decisions regarding add-on therapy may be made at any timepoint across the epilepsy disease course. The same criteria used in cohort 1 were applied to restrict the cohort to individuals without evidence of diabetes.

### Outcomes

For the antiepileptogenic model (cohorts 1), onset of the following dichotomous outcomes was assessed over the 10-year follow-up period:

- First recorded seizure (ICD-10-CM R56-coded).^45 46^
- First recorded epilepsy diagnosis (G40-coded).^45 46^
- First prescription of an ASM (listed in online supplemental 2), excluding gabapentinoids, as these are commonly prescribed for non-epilepsy indications.^45 46^

For the seizure symptom control model in epilepsy (cohort 2), outcomes included the next recorded seizure during follow-up (vs none). Consistent with our previous work in TriNetX,^39^ we also assessed the next potentially seizure-related events consisting of all-cause emergency department attendances and hospital admissions, falls, injuries and burns, considered both as individual outcomes and as a composite ‘net’ potentially seizure-related morbidity outcome. All-cause mortality was also assessed. The full coded outcome definitions are provided in online supplemental 3.

### Statistical analysis

Cohorts were propensity score matched for a comprehensive set of baseline covariates assessed at index. The baseline comparisons of characteristics employed χ^2^ tests for categorical variables and independent two-sample t-tests for continuous variables. Standardised mean differences (SMDs) were used to show the distribution of these characteristics among the cohorts and calculated as the difference in the means or proportions of a particular variable divided by the pooled estimate of standardised differences for that variable. Propensity scores were estimated using logistic regression (via scikit-learn). Matching was performed on a 1:1 basis using greedy nearest neighbour matching without replacement, with a calliper width of 0.1 pooled SD. Cohorts were randomly shuffled prior to matching to reduce order bias.^47 48^ An SMD threshold of ≤0.1 was used to indicate when covariates were well matched.^49^

The selection of the covariates was both informed by prior literature^42 50^ and guided by our clinical experience as a team specialising in neurology (GKM, WOP, NM and AGM), diabetes (UA) and public health (IEB). We used domain-specific knowledge to avoid adjusting for colliders and instrumental variables, focusing on adjusting confounders and competing exposures instead.^51 52^ We restricted propensity score matching to covariates measured prior to index to avoid adjusting for mediators, which can also distort effect estimates.^51 52^ Covariates included age, sex and ethnicity; Body Mass Index (BMI) and coded overweight/obesity diagnosis, PCOS and other metabolic disorders; HbA1c and blood glucose; external causes of morbidity (including falls, transport accidents, assaults and injuries); mental, behavioural and neurodevelopmental disorders (including alcohol and substance addiction); syncope and collapse; head injuries, cerebrovascular disease, malignancy, cerebral palsy, congenital malformations, other neurological disorders including chronic pain, migraine and sleep disorders, and general medical health (cardiovascular disease, respiratory disease, digestive disease (including alcoholic and non-alcoholic fatty liver disease), infectious disease (including COVID-19) and nutritional disorders). Relevant medication classes were also included (including antidepressants, sedatives/hypnotics, antipsychotics, central nervous system stimulants, opioid analgesics, antimicrobials, immunological agents, chemotherapy and cardiovascular medications). These covariates were selected as factors plausibly associated with both exposure allocation (metformin vs active weight-management comparators) and the outcomes of interest, including subsequent seizures or epilepsy diagnoses, seizure-related morbidities (emergency department attendances, hospital admissions, falls, injuries and burns) and all-cause mortality. Healthcare utilisation proxies derived from procedure groupings were also included to balance healthcare utilisation patterns between groups (evaluation and management, capturing outpatient activity, inpatient and emergency care; radiology; pathology/laboratory procedures; medicine services and procedures and surgery). For people with epilepsy (cohort 2), we also matched by type of epilepsy using the respective G40.- subgroups. Baseline ASM exposures were additionally included to balance ASM treatment patterns between the epilepsy groups. Full covariate definitions and results before and after propensity score matching are provided in online supplemental 4.

Follow-up commenced from 1 day after the index date and continued for up to 10 years. Analyses followed a pragmatic treatment-initiation framework, with follow-up retained regardless of subsequent treatment discontinuation to reduce the risk of informative censoring. Time-to-event analyses were performed using Cox proportional hazards regression to estimate HRs with 95% CIs. Kaplan–Meier curves were generated to visualise the probability of remaining free of recorded events over time and compared using log-rank tests.

As crude event counts do not account for variation in individual follow-up time, we estimated number needed to treat (NNT) using Kaplan–Meier survival probabilities at the end of the prespecified follow-up window. Cumulative risk at 10 years was calculated as 1− S(10 years), where S(t) is the Kaplan–Meier survival probability. Absolute risk differences (ARDs) were defined as the difference in 10 year cumulative risks between groups, and for statistically significant outcomes, we estimated the corresponding NNT as 1/ARD, anchored to the 10 year time window and interpreted alongside HRs. As TriNetX provides summary survival probabilities at the end of follow-up rather than time-specific estimates at multiple fixed intervals, ARD and NNT calculations were restricted to the 10 year endpoint.

To assess the potential impact of death as a competing risk for non-fatal outcomes, Aalen–Johansen cumulative incidence curves were generated in the unmatched cohorts and interpreted narratively alongside Cox models in which death was included as an outcome.

Participants were removed from the analysis (censored) after the last clinical data point in their record. All analyses were performed in the TriNetX LIVE platform using R4.0.2, Python V.3.7 and Java V.11.0.16.

## Results

### Cohort 1 (antiepileptogenesis)

We identified 12 991 people without evidence of diabetes who experienced an incident brain insult and were initiated within 3 years on either metformin (n=7325) or an active weight-management comparator (n=5666). They were drawn from 170 participating HCOs. Eighty-six per cent were based in the USA, and the remainder in non-US regions including Brazil, Italy, Spain, Taiwan and the UK. There were 8 746 759 electronic clinical data points across both cohorts. Before propensity score matching, mean BMI was in the obese range (BMI≥30 kg/m²) in both cohorts, at 31.4±7.5 kg/m² in the metformin group (consistent with the growing tendency to prescribe metformin for weight management) and 35.2±7.3 kg/m² in the active weight-management comparator group (SMD 0.517). Other prematched characteristics included a mean HbA1c of 5.2±0.5% and 5.2±0.3%, respectively (SMD 0.103). PCOS affected 7.9% and 3.5%, respectively (SMD 0.192). Cerebrovascular diseases overall (including ischaemic stroke and intracranial haemorrhages) affected 23.5% and 16.1% (SMD 0.187), head injury 68.6% and 82.3% (SMD 0.322) and malignancies (including brain tumours) 18.7% and 29.1% (SMD 0.247), respectively.

After propensity score matching, 2529 people were retained in each group. The matched groups were closely comparable, with a mean age of 45–46 years and approximately 70% women in both. Mean BMI remained in the obese range and slightly higher in the comparator group (34.0±7.1 kg/m²) than in the metformin group (32.1±8.2 kg/m²; SMD 0.244). All remaining covariates were well balanced, achieving SMDs<0.1 after matching (online supplemental 4). This included HbA1c levels, which remained similarly low in both groups (5.1±0.5% for metformin and 5.2%±0.3% for comparator), consistent with the predefined restriction excluding people with HbA1c≥5.7%. PCOS now affected 6.9% and 6.4% (SMD 0.019), cerebrovascular diseases 18.4% and 18.7% (SMD 0.007), head injury 78.1% and 77.8% (SMD 0.007) and malignancies 22.7% and 23.3% (SMD 0.015), respectively.

**Figure 1:**
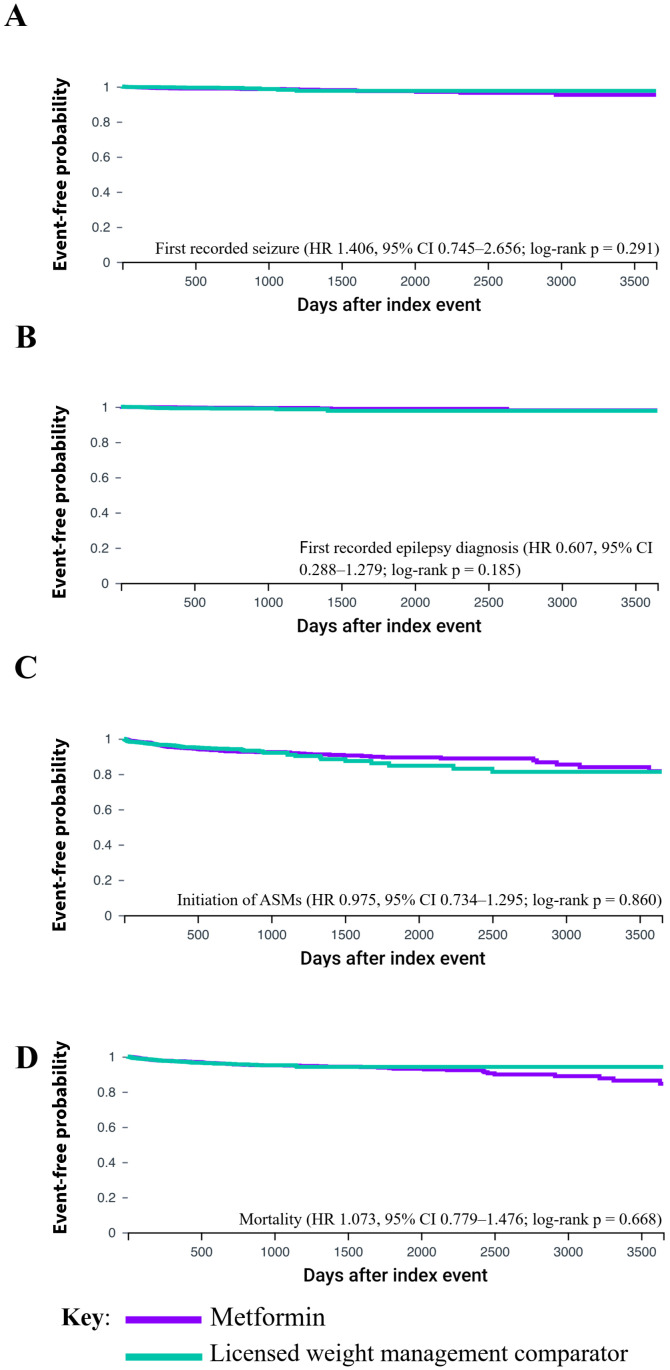
Kaplan–Meier plots for people without evidence of diabetes experiencing a brain insult treated with metformin (purple line) versus licensed weight management comparators (green line). (A) First recorded seizure, (B) first recorded epilepsy diagnosis, (C) first prescription of an antiseizure medication and (D) death.

There were no significant differences between metformin and active weight-management comparator in the risk of first recorded seizure (HR 1.406, 95% CI 0.745 to 2.656; log-rank p=0.291), first recorded epilepsy diagnosis (HR 0.607, 95% CI 0.288 to 1.279; log-rank p=0.185) or initiation of ASMs (HR 0.975, 95% CI 0.734 to 1.295; log-rank p=0.860)—see table 1 and figure 1A–C.

**Table 1:** Outcomes in patients managed with metformin or active weight-management comparators

Cohort	Outcome	Management	Patient count before matching	Patient count after matching	Patients with outcome	Survival probability at 10 years	HR (95% CI)	Log-rank p value
Cohort 1: people without diabetes experiencing a brain insult	First recorded seizure	Metformin	7325	2439*	29	95.24%	1.406 (0.745 to 2.656)	0.291
		Licensed weight loss therapy	5666	2441†	15	97.54%		
	First recorded epilepsy diagnosis	Metformin	7325	2436‡	13	97.93%	0.607 (0.288 to 1.279)	0.185
		Licensed weight loss therapy	5666	2443§	16	97.71%		
	First prescription of an ASM	Metformin	7325	2057¶	110	81.55%	0.975 (0.734 to 1.295)	0.860
		Licensed weight loss therapy	5666	2088**	89	81.17%		
	Death	Metformin	7325	2518††	91	84.54%	1.073 (0.779 to 1.476)	0.668
		Licensed weight loss therapy	5666	2523‡‡	67	94.07%		
Cohort 2: people with epilepsy and no evidence of diabetes who were treated with ASMs	One or more coded seizures	Metformin	2085	847	150	60.48%	**0.778 (0.624 to 0.971)**	**0.0257**
		Licensed weight loss therapy	1619	847	169	55.69%		
	One or more emergency department attendances or hospital admissions	Metformin	2085	847	180	53.36%	**0.696 (0.569 to 0.851)**	**0.0004**
		Licensed weight loss therapy	1619	847	206	32.20%		
	One or more falls, injuries or burns	Metformin	2085	847	90	73.48%	**0.677 (0.510 to 0.898)**	**0.0065**
		Licensed weight loss therapy	1619	847	106	62.74%		
	Net potentially seizure-related morbidity (any of the above outcomes)	Metformin	2085	847	319	28.28%	**0.729 (0.626 to 0.848)**	**< 0.0001**
		Licensed weight loss therapy	1619	847	362	12.44%		
	Death	Metformin	2085	847	29	87.45%	1.039 (0.594 to 1.816)	0.8937
		Licensed weight loss therapy	1619	846‡‡	22	94.11%		

Brain insult = ischaemic stroke, intracranial haemorrhage, head injury or brain tumour; licensed weight loss therapy = tirzepatide, liraglutide and semaglutide, orlistat (a lipase inhibitor) and bariatric surgery.

Results in bold correspond to statistically significant results.

* 90 patients in this cohort were excluded from results because they had the outcome prior to follow-up.

† 88 patients in this cohort were excluded from results because they had the outcome prior to follow-up.

‡ 93 patients in this cohort were excluded from results because they had the outcome prior to follow-up.

§ 86 patients in this cohort were excluded from results because they had the outcome prior to follow-up.

¶ 472 patients in this cohort were excluded from results because they had the outcome prior to follow-up.

** 441 patients in this cohort were excluded from results because they had the outcome prior to follow-up.

†† 11 patients in this cohort were excluded from results because they had the outcome prior to follow-up.

‡‡ 10 patients in this cohort were excluded from results because they had the outcome prior to follow-up.

§§ Statistically significant result.

ASM, antiseizure medication.

**Figure 2:**
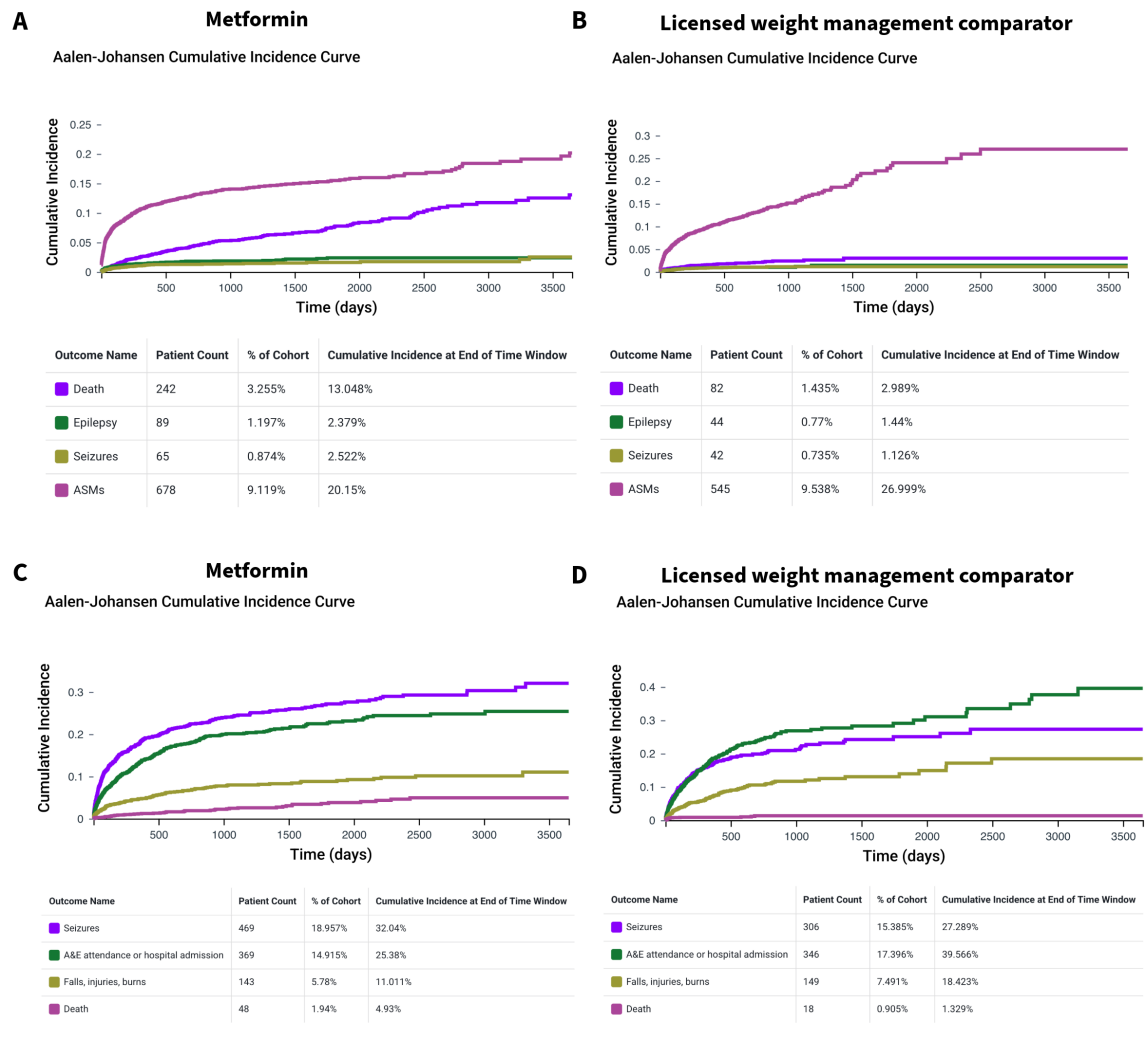
Cumulative incidence analyses of death against non-fatal outcomes. (A) People without evidence of diabetes experiencing a brain insult treated with metformin; (B) people without evidence of diabetes experiencing a brain insult treated with licensed weight management comparators; (C) people with epilepsy and no evidence of diabetes who were treated with ASMs and add-on metformin; (D) people with epilepsy and no evidence of diabetes who were treated with ASMs and add-on licensed weight management comparators. ASMs, antiseizure medication; A&E, accident and emergency.

In the unmatched competing-risk analysis, death was an important competing event over 10 years in both groups, more so in the metformin group (cumulative incidence 13%) than the active comparator group (cumulative incidence 3%; figure 2A,B). However, in the propensity-score-matched survival analyses, there was no evidence of a difference in all-cause mortality between groups (HR 1.073, 95% CI 0.779 to 1.476; log-rank p=0.668; figure 1D), suggesting that differential mortality was unlikely to explain the matched null associations observed for outcomes.

### Cohort 2 (seizure control)

We identified 3704 people with epilepsy and no evidence of diabetes who were treated with ASMs and commenced on metformin add-on therapy (n=2085) or active weight-management comparators as add-on (n=1619). They were drawn from 172 participating HCOs. Eighty-eight per cent were based in the USA, and the remainder in non-US regions including Brazil, Italy, Spain, Taiwan and the UK. There were 4 467 932 electronic clinical data points across both cohorts. Before propensity score matching, mean BMI was in the obese range (BMI≥30 kg/m²) in both cohorts, at 33.3±8.6 kg/m² in the metformin group (consistent with the growing tendency to prescribe metformin for weight management) and 36.1±8.0 kg/m² in the active weight-management comparator group. Other prematched characteristics included a mean HbA1c of 5.1±0.4% and 5.2±0.3%, respectively (SMD 0.077). PCOS affected 7.9% and 3.8%, respectively (SMD 0.177). Cerebrovascular diseases affected 7.9% and 9.3% (SMD 0.050), head injury 9.1% and 14.5% (SMD 0.170) and malignancies 13.1% and 21.9% (SMD 0.233), respectively. Epilepsy characteristics coded by type included unspecified epilepsy in 49.0% and 55.7% (SMD 0.136), focal epilepsy with complex partial seizures in 16.6% and 13.5% (SMD 0.085), generalised idiopathic epilepsy in 14.1% and 13.1% (SMD 0.028), focal epilepsy with simple partial seizures in 10.5% and 12.8% (SMD 0.072), other generalised epilepsy in 9.8% and 7.9% (SMD 0.066) and other epilepsy and recurrent seizures in 6.0% and 7.0% (SMD 0.041). The most commonly used ASMs were levetiracetam (38.6% and 38.4%; SMD 0.003), lamotrigine (29.6% and 28.8%; SMD 0.017), topiramate (23.1% and 31.3%; SMD 0.185) and valproate (21.0% and 12.7%; SMD 0.223), respectively.

After propensity score matching, 847 people were retained in each group. The matched groups were closely comparable, with a mean age of 41 years and approximately 70% women in both. All covariates were well balanced, achieving SMDs<0.1 after matching (online supplemental 4). This included an obese range mean BMI across both cohorts (34.2±9.1 kg/m² in the metformin group and 35.1±8.1 kg/m² in the comparator group; SMD 0.095), and HbA1c 5.1±0.5% and 5.2±0.3%, respectively; SMD 0.038. PCOS now affected 6.7% and 6.1%, respectively (SMD 0.024). Cerebrovascular diseases affected 7.9% and 9.3% (SMD 0.050), head injury 11.5% and 10.9% (SMD 0.019) and malignancies 17.5% and 15.9% (SMD 0.041), respectively. Epilepsy characteristics coded by type now consisted of unspecified epilepsy in 54.9% and 52.3% (SMD 0.052), focal epilepsy with complex partial seizures in 14.5% and 14.0% (SMD 0.013), generalised idiopathic epilepsy in 15.1% and 14.3% (SMD 0.023), focal epilepsy with simple partial seizures in 12.5% and 11.6% (SMD 0.029), other generalised epilepsy in 8.9% and 9.1% (SMD 0.008) and other epilepsy and recurrent seizures in 6.6% and 7.0% (SMD 0.014), respectively. The most commonly prescribed ASMs remained levetiracetam (40.5% and 40.0%; SMD 0.010), lamotrigine (31.4% and 30.6%; SMD 0.018), topiramate (25.6% and 27.5%; SMD 0.043) and valproate (15.7% and 15.3%; SMD 0.010).

### Potentially seizure-related morbidities

#### Coded seizures

**Figure 3:**
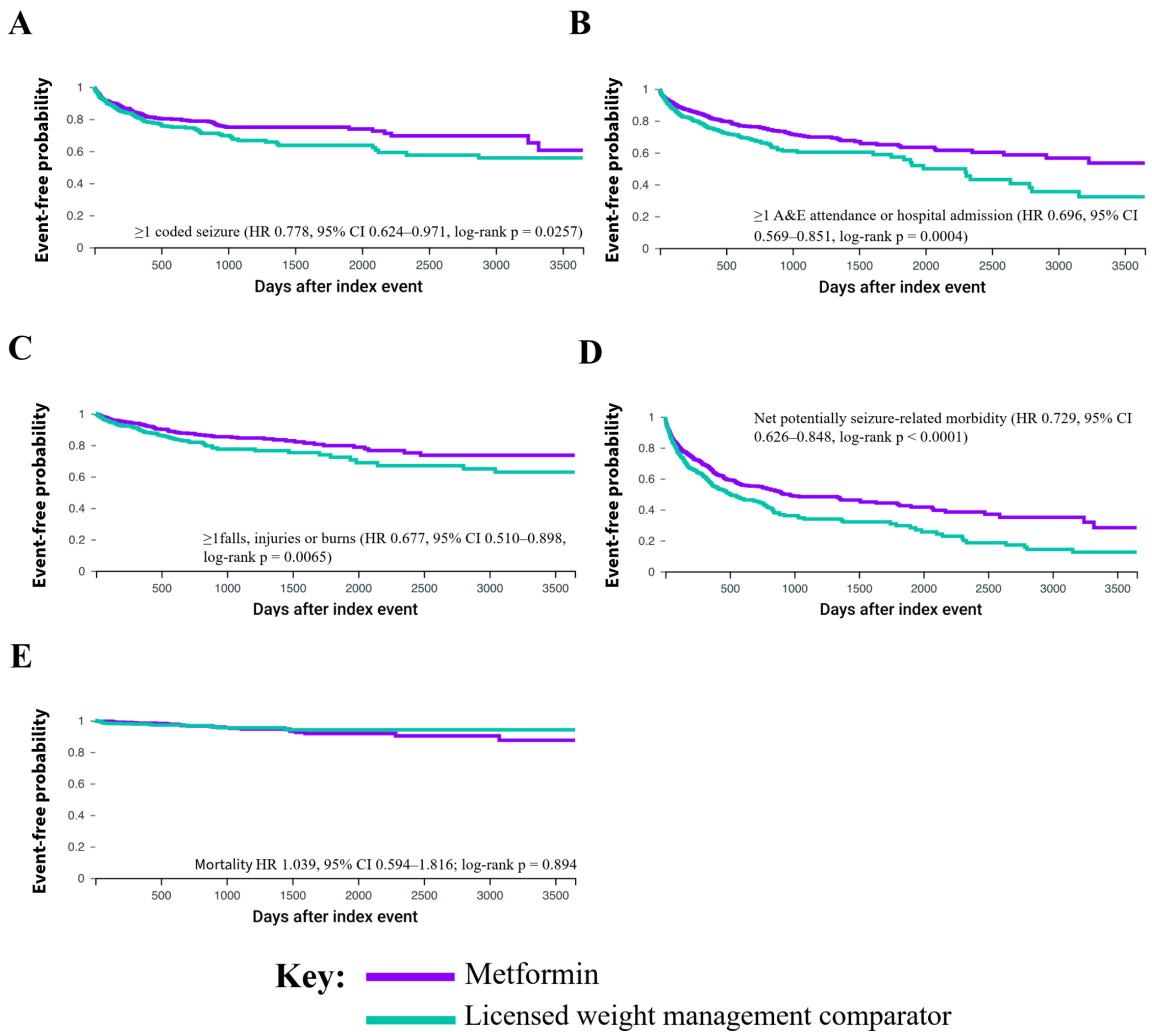
Kaplan–Meier plots for people with epilepsy and no evidence of diabetes who were treated with ASMs and metformin add-on (purple line) versus add-on licensed weight management comparators (green line). (A) Coded seizures; (B) emergency department attendances and hospital admissions; (C) falls, injuries, burns; (D) net potentially seizure-related morbidity (composite of A–C) and (E) death. ASM, antiseizure medication; A&E, accident and emergency.

Metformin treatment was associated with a significantly lower hazard of 22.2% for experiencing one or more coded seizures over 10 years (HR 0.778, 95% CI 0.624 to 0.971, log-rank p=0.0257; table 1, figure 3A). At 10 years, Kaplan–Meier survival probability was 60.48% in the metformin group and 55.69% in the active comparator group, corresponding to an absolute risk reduction of 4.79% and an approximate NNT of 21.

#### Emergency department attendance and hospital admission

Metformin treatment was associated with a significantly lower hazard of 30.4% for experiencing one or more emergency department attendances or hospital admissions over 10 years (HR 0.696, 95% CI 0.569 to 0.851, log-rank p=0.0004; table 1, figure 3B). At 10 years, Kaplan–Meier survival probability was 53.36% in the metformin group and 32.20% in the active comparator group, corresponding to an absolute risk reduction of 21.2% and an approximate NNT of 5.

#### Falls, injuries and burns

Metformin treatment was associated with a significantly lower hazard of 32.3% for experiencing one or more falls, injuries or burns over 10 years (HR 0.677, 95% CI 0.510 to 0.898, log-rank p=0.0065; table 1, figure 3C). At 10 years, Kaplan–Meier survival probability was 73.48% in the metformin group and 62.74% in the active comparator group, corresponding to an absolute risk reduction of 10.7% and an approximate NNT of 10.

#### Net potentially seizure-related morbidity (any of the above outcomes)

Metformin treatment was associated with a significantly lower hazard of 27.1% for experiencing one or more potentially seizure-related morbidity events over 10 years (HR 0.729, 95% CI 0.626 to 0.848, log-rank p<0.0001; table 1, figure 3D). At 10 years, Kaplan–Meier survival probability was 28.28% in the metformin group and 12.44% in the active comparator group, corresponding to an absolute risk reduction of 15.8% and an approximate NNT of 6.

### All-cause deaths

There were no significant differences between metformin and the active weight-management comparator in the risk of death (HR 1.039, 95% CI 0.594 to 1.816; log-rank p=0.894; table 1, figure 3E), suggesting an overall balance in background health status between the groups.

In the unmatched competing-risk analysis (figure 2C,D), death was an infrequent competing event in both groups (cumulative incidence 4.93% with metformin versus 1.33% with the active weight-management comparator at 10 years). In contrast, non-fatal outcomes accrued more commonly, including coded seizures (32.04% vs 27.29%, respectively), accident and emergency attendance or hospital admission (25.38% vs 39.57%), and falls, injuries or burns (11.01% vs 18.42%). Overall, these findings suggest that the observed reduction in the potentially seizure-related morbidity outcomes with metformin is unlikely to be explained by differential competing mortality.

## Discussion

In this large retrospective cohort study using real-world healthcare data, we made two key findings:

1. Among people without evidence of diabetes experiencing an incident brain insult (ischaemic stroke, intracranial haemorrhage, head injury or brain tumour), there was no statistical indication that metformin is associated with epilepsy prevention (antiepileptogenesis).
2. Among people with epilepsy without evidence of diabetes who were treated with ASMs, metformin add-on therapy was associated with a significant reduction in potentially seizure-related morbidity. This was reflected by fewer coded seizures recorded in healthcare settings, fewer emergency department attendances and hospital admissions and fewer accidents (falls, injuries, burns) that are commonly seizure-related. The observed reduction in potentially seizure-related morbidity in those on metformin translated to an approximate NNT of 6 to prevent one morbidity event over 10 years. This effect was observed largely in the context of obesity.

A competing risk from mortality is unlikely to account for the observed reduction in potentially seizure-related morbidity that was seen with metformin in people with epilepsy. The competing-risk analyses indicated that death was an infrequent competing event relative to the non-fatal morbidity outcomes, and there was no difference in mortality between treatment groups after propensity score matching. This suggests that baseline health status was comparable once measured differences between cohorts had been accounted for. Nevertheless, we could not account for the influence of any unmeasured confounders. The large number of covariates included in our analysis may have reduced this risk to some extent, as some will be correlated with potential unmeasured confounders. Future work could aim to further address unmeasured confounding using methods such as E-values and instrumental variable analyses.^53 54^

The results of this study align more closely with preclinical evidence suggesting antiseizure effects of metformin, rather than antiepileptogenic effects.^11 12 15–17^ Uniquely, we restricted the analysis to individuals without evidence of diabetes. This was defined not only by the absence of diagnostic codes, but also by serum glucose and HbA1c thresholds, and by the absence of exposure to other antidiabetic drugs outside the treatments of interest. This approach aimed to test whether any observed signal could plausibly be present outside glycaemic control. This should be interpreted cautiously, as the absence of coded evidence of diabetes in health data does not necessarily equate to the absence of diabetes. However, undetected diabetes is unlikely to have fully accounted for the observed signal, as any misclassification would be expected to occur similarly across the treatment arms. This is consistent with the postmatching balance in BMI, serum glucose and HbA1c in the epilepsy cohort. Moreover, the active comparator group consisted of both licensed antidiabetic and weight-management therapies, reducing the likelihood that the findings simply reflect metformin treating diabetes and weight loss. The active comparator even included bariatric surgery, which achieves greater and more sustained weight reduction than any pharmacotherapy.^55^ The use of an active comparator design alongside additional adjustment for healthcare utilisation patterns also reduced the likelihood that the findings simply reflect differences in access to or intensity of clinical care. Therefore, overall, the observed association between metformin add-on therapy and reduced potentially seizure-related morbidity in established epilepsy in our study may warrant further investigation and validation in external data sets. If replicated, these findings could support consideration of metformin as a candidate adjunctive therapy in further epilepsy clinical trials, consistent with translational precedents such as fenfluramine, which was repurposed from weight management to epilepsy treatment.^33–37^ The composite net potentially seizure-related morbidity outcome may provide the most clinically representative endpoint for such trials. Using the observed HR of 0.729 and the Kaplan–Meier survival probabilities at 10 years in this cohort, an illustrative log-rank based calculation suggests that approximately 315 events would be required, corresponding to around 400 participants (about 200 per arm) with 10 year follow-up (online supplemental 5).^56^

Interpretation of the lack of antiepileptogenesis in this study should take into account the 24 month exposure window used in this real-world design, following the incident brain insult. Metformin was rarely initiated contemporaneously with the insult, limiting our ability to evaluate effects within the earliest postinsult period when seizure risk is typically highest. In addition, we included a heterogeneous mix of brain insults to maximise sample size, which may have reduced sensitivity to detect insult-specific antiepileptogenic effects. Further work should be undertaken to explore whether there remains any residual antiepileptogenic effect early on following specific brain insults with metformin exposure.

Approximately 55% of people with epilepsy are overweight or obese.^57^ The excess burden of obesity in epilepsy is multifactorial, reflecting a combination of refractory disease, reduced physical activity, adverse sociodemographic and mental health factors and the metabolic effects of certain ASMs, particularly valproate.^25 26 58^ However, obesity in epilepsy is not fully explained by medication exposure or socioeconomic confounding alone.^23 26 57 59 60^ Mendelian randomisation provides evidence for a bidirectional relationship between obesity and epilepsy, with higher BMI and specific adiposity measures, including waist and hip circumference, increasing the risk of certain epilepsy subtypes and epilepsy in turn influencing measures of adiposity.^59^ Cross-sectional population data also demonstrate that elevated Body Roundness Index, a marker of central adiposity, is associated with a higher prevalence of epilepsy.^24^ Despite this emerging evidence, further large-scale prospective studies are required to clarify causal pathways and inform targeted interventions between obesity and epilepsy. In the interim, guidance from The Obesity Society supports a pragmatic approach involving regular BMI monitoring and weight-informed rationalisation of ASMs in people with epilepsy.^58^ Further should also explore whether metabolic changes contribute to the relationship between metformin and seizures by examining longitudinal changes in BMI following treatment initiation and conducting mediation analyses to assess whether BMI changes lie on the causal pathway between treatment exposure and seizure outcomes.

Although many people will be familiar with metformin given how common diabetes is, few will have ever considered it for epilepsy. While a growing body of evidence in animal models supports metformin’s antiepileptogenic and antiseizure properties,^10–14^ translating these findings into clinical trials has remained challenging, contributing to the lack of robust in-human evidence in this area. Our work highlights the role of health data research in bridging the gap between evidence from animal models and its translation into clinical practice. This is a growing area. A recently published study used a health data discovery approach within the US Department of Veterans Affairs databases to map out the effectiveness of GLP-1 receptor antagonists compared with usual care for 175 comparator health outcomes including seizures in people with diabetes.^50^ The authors adjusted for a multitude of covariates including metformin, and demonstrated reduced risk of seizures with GLP-1RA exposure: HR 0.90, 95% CI 0.85 to 0.95. GLP-1RAs were part of the active comparator arm in our study, which could, in theory, have actually diluted the magnitude of any effect seen with metformin. Nevertheless, taken together with our findings, it suggests positive signals are emerging along the epilepsy drug discovery path, with health data approaches offering a deliverable framework to support this journey.

### Strengths and limitations

Our study is strengthened by being an analysis of real-world healthcare data extracted directly from clinical care.^40 41^ The results are generalisable to both neurology and general medicine and suggest need for a more holistic, multidisciplinary approach to epilepsy care. The study is further strengthened by its large size and ability to adjust for a wide range of baseline covariates, allowing for fair comparison between groups. As expected in real-world data sets, several baseline characteristics differed between groups. Propensity score matching was chosen to address these imbalances. The use of propensity score matching is a strength because this method emulates the effect of randomisation by matching individuals with similar probabilities of treatment based on their observed baseline characteristics.^61^ Future work should seek to complement our approach by inverse probability weighting the covariates at baseline (another way to emulate randomisation). Marginal structural models or nested sequential trial emulations could also be considered to account for subsequent time-varying confounding.^54 62–64^ Such methods are not currently available in the TriNetX LIVE platform we used for analysis, but could be applied on a raw data extract in future, ideally within the causal framework of a target trial emulation.^62 63 65^ Within this context, future studies could also explore whether metabolic changes contribute to the associations observed with metformin in our study by examining longitudinal changes in BMI after initiation of metformin and other weight-management therapies, as part of a mediation analysis.^66^ This would determine whether BMI changes lie on the causal pathway between treatment exposure and seizure outcomes. Similar mediation analyses could also be applied to cerebrovascular and other potential mechanistic pathways through which metformin may exert effects.^66^ The idea that metabolic and vascular changes associated with weight-modifying therapies may plausibly influence seizure outcomes represents an interesting avenue for future mechanistic and prospective investigation, particularly given the successful repurposing of fenfluramine from weight management to epilepsy.^31–37^

Our study was limited to capturing potentially seizure-related morbidity as the full spectrum of seizures (complete seizure counts and their severity) cannot be fully or accurately captured in any setting other than perhaps inpatient video telemetry, which is not pragmatic even for a clinical trial.^67^ Although coded seizures do not capture all seizure activity and are subject to diagnostic misclassification, seizures that prompt healthcare contact and are subsequently (and correctly) coded can be clinically meaningful in their own right as they may reflect seizure severity or other features of a seizure that drive a patient to make clinical contact. Therefore, coded seizure signal should not be simply discarded in health data analyses. However, it should be interpreted cautiously, recognising that it does not reflect total seizure burden and may have inaccuracy. Indeed, there remain no validated coding strategies to capture seizure symptoms in health data, so future studies are still needed to clarify the interpretation of any outcome attributing seizures to, for example, the ICD-10 code for seizure (R56).^45 46^ For this reason, we complemented coded seizures with additional potentially seizure-related morbidity outcomes: emergency department attendances and hospital admissions, falls, injuries, and burns.^39^ We also mitigated diagnostic inaccuracy in coding as far as possible by ensuring that we used codes previously validated as being able to reliably identify epilepsy within healthcare datasets.^45 46 68^ This was further offset by our use of large, well-matched cohorts, between which coding errors are likely to be distributed evenly.

Although our exposure estimand was a pragmatic treatment-initiation (intention-to-treat) policy, commencing metformin versus licensed weight-management comparators, our study was ultimately limited by estimating a per-protocol-type effect. This is because postbaseline information was required to confirm ongoing eligibility (continued absence of diabetes progression and no exposure to competing antidiabetic or weight-management therapies outside the treatments of interest). While this approach improved exposure specificity and supported the intended biological interpretation, it may have introduced immortal time bias and reduced generalisability to real-world settings, where many individuals with obesity subsequently develop diabetes. Future work using raw data extracts should enable more robust estimation of intention-to-treat effects.

Further limitations include that we were unable to account for the frequency or dose of metformin exposure. Dose–response should be assessed in future studies. An assumption was also made that patients who were prescribed metformin were adherent to the treatment, but it was not possible to test this. Nevertheless, any bias resultant from this should also have been equally distributed among the large and well-matched cohorts studied.

In conclusion, this large propensity-score-matched study of real-world healthcare data suggests that metformin, the most widely available treatment for T2DM globally, may be associated with reduced seizure-related morbidity in people with epilepsy without evidence of diabetes, particularly in the context of comorbid obesity. These findings are hypothesis-generating and require external validation.

## Supplementary material

10.1136/jnnp-2025-337861 online supplemental file 1

## Data Availability

Data may be obtained from a third party and are not publicly available.
