# Supplementary material for: Evaluation of metformin for epilepsy prevention or seizure control in people without evidence of diabetes: retrospective cohort study
Source: J Neurol Neurosurg Psychiatry. 2026 May 13;97(8):e337861. doi: 10.1136/jnnp-2025-337861 (PMC13479215; doi:10.1136/jnnp-2025-337861)
Supplement: Supplementary file 1 [file jnnp-97-8-s001.docx]

Supplementary Material

[Supplement 1: Study cohorts 2](#_Toc219701742)

[Cohort 1 (antiepileptogenesis) 2](#_Toc219701743)

[Metformin group 2](#_Toc219701744)

[Active weight-management comparator group 3](#_Toc219701745)

[Cohort 2 (seizure control) 4](#_Toc219701746)

[Metformin group 4](#_Toc219701747)

[Active weight-management comparator group 5](#_Toc219701748)

[Case-ascertainment accuracy 7](#_Toc219701749)

[Supplement 2: Antiseizure medications considered 7](#_Toc219701750)

[Supplement 3: Coded outcome definitions 9](#_Toc219701751)

[Cohort 1 (antiepileptogenesis) 9](#_Toc219701752)

[Cohort 2 (seizure control) 9](#_Toc219701753)

[Supplement 4: Propensity score matching results 12](#_Toc219701754)

[Cohort 1 (antiepileptogenesis) 12](#_Toc219701755)

[Cohort 2 (seizure control) 20](#_Toc219701756)

[Supplement 5: Illustrative sample size calculation for a future trial 31](#_Toc219701757)

# **Supplement 1: Study cohorts**

## Cohort 1 (antiepileptogenesis)

### Metformin group

The following logic (combining groups 1, 2 and 3) was used to build a cohort of people without evidence of diabetes experiencing an incident brain insult (ischaemic stroke, intracranial haemorrhage, head injury, or brain tumour) who were commenced on metformin.

| Group 1 | | | | | |
| --- | --- | --- | --- | --- | --- |
|  | **No diabetes** | | | | |
|  | cannot have |  | diagnosis | UMLS:ICD10CM:E08-E13 | Diabetes mellitus |
|  |  | or | diagnosis | UMLS:ICD10CM:R73.0 | Abnormal glucose |
|  |  | or | laboratory | TNX:9037 | Hemoglobin A1c/Hemoglobin.total in Blood (at least 5.70 %) |
|  |  | or | laboratory | TNX:9025 | Glucose [Mass/volume] in Serum, Plasma or Blood (at least 100.00 mg/dL) |
|  |  | or | medication | NLM:RXNORM:857974 | saxagliptin |
|  |  | or | medication | NLM:RXNORM:84108 | rosiglitazone |
|  |  | or | medication | NLM:RXNORM:73044 | repaglinide |
|  |  | or | medication | NLM:RXNORM:593411 | sitagliptin |
|  |  | or | medication | NLM:RXNORM:4821 | glipizide |
|  |  | or | medication | NLM:RXNORM:4815 | glyburide |
|  |  | or | medication | NLM:RXNORM:33738 | pioglitazone |
|  |  | or | medication | NLM:RXNORM:30009 | miglitol |
|  |  | or | medication | NLM:RXNORM:274332 | nateglinide |
|  |  | or | medication | NLM:RXNORM:2638675 | sotagliflozin |
|  |  | or | medication | NLM:RXNORM:2627044 | bexagliflozin |
|  |  | or | medication | NLM:RXNORM:25789 | glimepiride |
|  |  | or | medication | NLM:RXNORM:1992672 | ertugliflozin |
|  |  | or | medication | NLM:RXNORM:16681 | acarbose |
|  |  | or | medication | NLM:RXNORM:1545653 | empagliflozin |
|  |  | or | medication | NLM:RXNORM:1488564 | dapagliflozin |
|  |  | or | medication | NLM:RXNORM:1373458 | canagliflozin |
|  |  | or | medication | NLM:RXNORM:1368001 | alogliptin |
|  |  | or | medication | NLM:RXNORM:1100699 | linagliptin |
|  |  | or | medication | NLM:RXNORM:10635 | tolbutamide |
|  |  | or | medication | NLM:RXNORM:10633 | tolazamide |
|  |  | or | medication | NLM:VA:HS509 | HYPOGLYCEMIC AGENTS,OTHER |
|  |  | or | medication | NLM:VA:HS503 | ANTIHYPOGLYCEMICS |
|  |  | or | medication | NLM:VA:HS501 | INSULIN |
|  |  | or | medication | NLM:RXNORM:60548 | exenatide |
|  | date constraint | | The terms in this group occurred at any time | | |
| Group 2 | | | | | |
|  | **no other study weight loss therapy** | | | | |
|  | cannot have |  | medication | NLM:RXNORM:37925 | orlistat |
|  |  | or | procedure | UMLS:CPT:1007385 | Bariatric Surgery Procedures |
|  | date constraint | | The terms in this group occurred at any time | | |
| Group 3 | | | | | |
|  | **Group 3A seizure risk factors** | | | | |
|  | must have | any of | diagnosis | UMLS:ICD10CM:I60 | Nontraumatic subarachnoid hemorrhage |
|  |  |  | diagnosis | UMLS:ICD10CM:I61 | Nontraumatic intracerebral hemorrhage |
|  |  |  | diagnosis | UMLS:ICD10CM:I62 | Other and unspecified nontraumatic intracranial hemorrhage |
|  |  |  | diagnosis | UMLS:ICD10CM:I63 | Cerebral infarction |
|  |  |  | diagnosis | UMLS:ICD10CM:C71 | Malignant neoplasm of brain |
|  |  |  | diagnosis | UMLS:ICD10CM:D32 | Benign neoplasm of meninges |
|  |  |  | diagnosis | UMLS:ICD10CM:C79.3 | Secondary malignant neoplasm of brain and cerebral meninges |
|  |  |  | diagnosis | UMLS:ICD10CM:S00-S09 | Injuries to the head |
|  | date constraint | | The terms in this group occurred at any time | | |
|  | event relationship | | The first instance of metformin occurred within 3 years on or after the first instance of seizure risk factors | | |
|  | **Group 3B metformin** | | | | |
|  | must have |  | medication | NLM:RXNORM:6809 | metformin |

### Active weight-management comparator group

The following logic (combining groups 1, 2 and 3) was used to build a cohort of people without evidence of diabetes experiencing an incident brain insult (ischaemic stroke, intracranial haemorrhage, head injury, or brain tumour) who were commenced on licensed weight-management therapies as active comparators: tirzepatide, liraglutide and semaglutide (GLP-1 receptor agonists that are also licensed treatments for T2DM), orlistat (a lipase inhibitor), and bariatric surgery.

| Group 1 | | | | | |
| --- | --- | --- | --- | --- | --- |
|  | **No diabetes** | | | | |
|  | cannot have |  | diagnosis | UMLS:ICD10CM:E08-E13 | Diabetes mellitus |
|  |  | or | diagnosis | UMLS:ICD10CM:R73.0 | Abnormal glucose |
|  |  | or | laboratory | TNX:9037 | Hemoglobin A1c/Hemoglobin.total in Blood (at least 5.70 %) |
|  |  | or | laboratory | TNX:9025 | Glucose [Mass/volume] in Serum, Plasma or Blood (at least 100.00 mg/dL) |
|  |  | or | medication | NLM:RXNORM:6809 | metformin |
|  |  | or | medication | NLM:RXNORM:857974 | saxagliptin |
|  |  | or | medication | NLM:RXNORM:84108 | rosiglitazone |
|  |  | or | medication | NLM:RXNORM:73044 | repaglinide |
|  |  | or | medication | NLM:RXNORM:593411 | sitagliptin |
|  |  | or | medication | NLM:RXNORM:4821 | glipizide |
|  |  | or | medication | NLM:RXNORM:4815 | glyburide |
|  |  | or | medication | NLM:RXNORM:33738 | pioglitazone |
|  |  | or | medication | NLM:RXNORM:30009 | miglitol |
|  |  | or | medication | NLM:RXNORM:274332 | nateglinide |
|  |  | or | medication | NLM:RXNORM:2638675 | sotagliflozin |
|  |  | or | medication | NLM:RXNORM:2627044 | bexagliflozin |
|  |  | or | medication | NLM:RXNORM:25789 | glimepiride |
|  |  | or | medication | NLM:RXNORM:1992672 | ertugliflozin |
|  |  | or | medication | NLM:RXNORM:16681 | acarbose |
|  |  | or | medication | NLM:RXNORM:1545653 | empagliflozin |
|  |  | or | medication | NLM:RXNORM:1488564 | dapagliflozin |
|  |  | or | medication | NLM:RXNORM:1373458 | canagliflozin |
|  |  | or | medication | NLM:RXNORM:1368001 | alogliptin |
|  |  | or | medication | NLM:RXNORM:1100699 | linagliptin |
|  |  | or | medication | NLM:RXNORM:10635 | tolbutamide |
|  |  | or | medication | NLM:RXNORM:10633 | tolazamide |
|  |  | or | medication | NLM:VA:HS501 | INSULIN |
|  |  | or | medication | NLM:RXNORM:60548 | exenatide |
|  |  | or | medication | NLM:RXNORM:139953 | pramlintide |
|  |  | or | medication | NLM:RXNORM:1440051 | lixisenatide |
|  |  | or | medication | NLM:RXNORM:1551291 | dulaglutide |
|  |  | or | medication | NLM:RXNORM:2621880 | teplizumab |
|  | date constraint | | The terms in this group occurred at any time | | |
| Group 2 | | | | | |
|  | **Group 2A seizure risk factors** | | | | |
|  | must have | any of | diagnosis | UMLS:ICD10CM:I60 | Nontraumatic subarachnoid hemorrhage |
|  |  |  | diagnosis | UMLS:ICD10CM:I61 | Nontraumatic intracerebral hemorrhage |
|  |  |  | diagnosis | UMLS:ICD10CM:I62 | Other and unspecified nontraumatic intracranial hemorrhage |
|  |  |  | diagnosis | UMLS:ICD10CM:I63 | Cerebral infarction |
|  |  |  | diagnosis | UMLS:ICD10CM:C71 | Malignant neoplasm of brain |
|  |  |  | diagnosis | UMLS:ICD10CM:D32 | Benign neoplasm of meninges |
|  |  |  | diagnosis | UMLS:ICD10CM:C79.3 | Secondary malignant neoplasm of brain and cerebral meninges |
|  |  |  | diagnosis | UMLS:ICD10CM:S00-S09 | Injuries to the head |
|  | date constraint | | The terms in this group occurred at any time | | |
|  | event relationship | | The first instance of weight loss therapy (tirzepatide/liraglutide/semaglutide/orlistat/bariatric surgery) occurred within 3 years on or after the first instance of seizure risk factors | | |
|  | **Group 2B weight loss therapy (tirzepatide/liraglutide/semaglutide/orlistat/bariatric surgery)** | | | | |
|  | must have | any of | medication | NLM:RXNORM:2601723 | tirzepatide |
|  |  |  | medication | NLM:RXNORM:475968 | liraglutide |
|  |  |  | medication | NLM:RXNORM:1991302 | semaglutide |
|  |  |  | medication | NLM:RXNORM:37925 | orlistat |
|  |  |  | procedure | UMLS:CPT:1007385 | Bariatric Surgery Procedures |

## Cohort 2 (seizure control)

### Metformin group

The following logic (combining groups 1, 2 and 3) was used to build a cohort of people with epilepsy and no evidence of diabetes who were treated with ASMs and commenced on metformin add-on therapy.

| Group 1 | | | | | |
| --- | --- | --- | --- | --- | --- |
|  | **No diabetes** | | | | |
|  | cannot have |  | diagnosis | UMLS:ICD10CM:E08-E13 | Diabetes mellitus |
|  |  | or | diagnosis | UMLS:ICD10CM:R73.0 | Abnormal glucose |
|  |  | or | laboratory | TNX:9037 | Hemoglobin A1c/Hemoglobin.total in Blood (at least 5.70 %) |
|  |  | or | laboratory | TNX:9025 | Glucose [Mass/volume] in Serum, Plasma or Blood (at least 100.00 mg/dL) |
|  |  | or | medication | NLM:RXNORM:857974 | saxagliptin |
|  |  | or | medication | NLM:RXNORM:84108 | rosiglitazone |
|  |  | or | medication | NLM:RXNORM:73044 | repaglinide |
|  |  | or | medication | NLM:RXNORM:593411 | sitagliptin |
|  |  | or | medication | NLM:RXNORM:4821 | glipizide |
|  |  | or | medication | NLM:RXNORM:4815 | glyburide |
|  |  | or | medication | NLM:RXNORM:33738 | pioglitazone |
|  |  | or | medication | NLM:RXNORM:30009 | miglitol |
|  |  | or | medication | NLM:RXNORM:274332 | nateglinide |
|  |  | or | medication | NLM:RXNORM:2638675 | sotagliflozin |
|  |  | or | medication | NLM:RXNORM:2627044 | bexagliflozin |
|  |  | or | medication | NLM:RXNORM:25789 | glimepiride |
|  |  | or | medication | NLM:RXNORM:1992672 | ertugliflozin |
|  |  | or | medication | NLM:RXNORM:16681 | acarbose |
|  |  | or | medication | NLM:RXNORM:1545653 | empagliflozin |
|  |  | or | medication | NLM:RXNORM:1488564 | dapagliflozin |
|  |  | or | medication | NLM:RXNORM:1373458 | canagliflozin |
|  |  | or | medication | NLM:RXNORM:1368001 | alogliptin |
|  |  | or | medication | NLM:RXNORM:1100699 | linagliptin |
|  |  | or | medication | NLM:RXNORM:10635 | tolbutamide |
|  |  | or | medication | NLM:RXNORM:10633 | tolazamide |
|  |  | or | medication | NLM:VA:HS509 | HYPOGLYCEMIC AGENTS,OTHER |
|  |  | or | medication | NLM:VA:HS503 | ANTIHYPOGLYCEMICS |
|  |  | or | medication | NLM:VA:HS501 | INSULIN |
|  |  | or | medication | NLM:RXNORM:60548 | exenatide |
|  | date constraint | | The terms in this group occurred at any time | | |
| Group 2 | | | | | |
|  | **no other study weight loss therapy** | | | | |
|  | cannot have |  | medication | NLM:RXNORM:37925 | orlistat |
|  |  | or | procedure | UMLS:CPT:1007385 | Bariatric Surgery Procedures |
|  | date constraint | | The terms in this group occurred at any time | | |
| Group 3 | | | | | |
|  | **Group 3A seizure or epilepsy on an ASM** | | | | |
|  | must have | any of | diagnosis | UMLS:ICD10CM:R56 | Convulsions, not elsewhere classified |
|  |  |  | diagnosis | UMLS:ICD10CM:G40 | Epilepsy and recurrent seizures |
|  |  | and any of | medication | NLM:RXNORM:114477 | levetiracetam |
|  |  |  | medication | NLM:RXNORM:21241 | clobazam |
|  |  |  | medication | NLM:RXNORM:1356552 | perampanel |
|  |  |  | medication | NLM:RXNORM:1482502 | eslicarbazepine |
|  |  |  | medication | NLM:RXNORM:14851 | vigabatrin |
|  |  |  | medication | NLM:RXNORM:1739745 | brivaracetam |
|  |  |  | medication | NLM:RXNORM:2002 | carbamazepine |
|  |  |  | medication | NLM:RXNORM:2045371 | cannabidiol |
|  |  |  | medication | NLM:RXNORM:2054968 | stiripentol |
|  |  |  | medication | NLM:RXNORM:2265690 | cenobamate |
|  |  |  | medication | NLM:RXNORM:24812 | felbamate |
|  |  |  | medication | NLM:RXNORM:2604689 | ganaxolone |
|  |  |  | medication | NLM:RXNORM:28439 | lamotrigine |
|  |  |  | medication | NLM:RXNORM:31914 | tiagabine |
|  |  |  | medication | NLM:RXNORM:32624 | oxcarbazepine |
|  |  |  | medication | NLM:RXNORM:38404 | topiramate |
|  |  |  | medication | NLM:RXNORM:39998 | zonisamide |
|  |  |  | medication | NLM:RXNORM:40254 | valproate |
|  |  |  | medication | NLM:RXNORM:4135 | ethosuximide |
|  |  |  | medication | NLM:RXNORM:4328 | fenfluramine |
|  |  |  | medication | NLM:RXNORM:47858 | methsuximide |
|  |  |  | medication | NLM:RXNORM:623400 | lacosamide |
|  |  |  | medication | NLM:RXNORM:69036 | rufinamide |
|  |  |  | medication | NLM:RXNORM:72236 | fosphenytoin |
|  |  |  | medication | NLM:RXNORM:8183 | phenytoin |
|  |  |  | medication | NLM:RXNORM:8691 | primidone |
|  |  |  | medication | NLM:RXNORM:8134 | phenobarbital |
|  | date constraint | | The terms in this group occurred at any time | | |
|  | event relationship | | The first instance of metformin occurred on or after the first instance of seizure or epilepsy on an ASM | | |
|  | **Group 3B metformin** | | | | |
|  | must have |  | medication | NLM:RXNORM:6809 | metformin |

### Active weight-management comparator group

The following logic (combining groups 1, 2 and 3) was used to build a cohort of people with epilepsy and no evidence of diabetes who were treated with active weight-management comparators as add-on.

| Group 1 | | | | | |
| --- | --- | --- | --- | --- | --- |
|  | **No diabetes** | | | | |
|  | cannot have |  | diagnosis | UMLS:ICD10CM:E08-E13 | Diabetes mellitus |
|  |  | or | diagnosis | UMLS:ICD10CM:R73.0 | Abnormal glucose |
|  |  | or | laboratory | TNX:9037 | Hemoglobin A1c/Hemoglobin.total in Blood (at least 5.70 %) |
|  |  | or | laboratory | TNX:9025 | Glucose [Mass/volume] in Serum, Plasma or Blood (at least 100.00 mg/dL) |
|  |  | or | medication | NLM:RXNORM:6809 | metformin |
|  |  | or | medication | NLM:RXNORM:857974 | saxagliptin |
|  |  | or | medication | NLM:RXNORM:84108 | rosiglitazone |
|  |  | or | medication | NLM:RXNORM:73044 | repaglinide |
|  |  | or | medication | NLM:RXNORM:593411 | sitagliptin |
|  |  | or | medication | NLM:RXNORM:4821 | glipizide |
|  |  | or | medication | NLM:RXNORM:4815 | glyburide |
|  |  | or | medication | NLM:RXNORM:33738 | pioglitazone |
|  |  | or | medication | NLM:RXNORM:30009 | miglitol |
|  |  | or | medication | NLM:RXNORM:274332 | nateglinide |
|  |  | or | medication | NLM:RXNORM:2638675 | sotagliflozin |
|  |  | or | medication | NLM:RXNORM:2627044 | bexagliflozin |
|  |  | or | medication | NLM:RXNORM:25789 | glimepiride |
|  |  | or | medication | NLM:RXNORM:1992672 | ertugliflozin |
|  |  | or | medication | NLM:RXNORM:16681 | acarbose |
|  |  | or | medication | NLM:RXNORM:1545653 | empagliflozin |
|  |  | or | medication | NLM:RXNORM:1488564 | dapagliflozin |
|  |  | or | medication | NLM:RXNORM:1373458 | canagliflozin |
|  |  | or | medication | NLM:RXNORM:1368001 | alogliptin |
|  |  | or | medication | NLM:RXNORM:1100699 | linagliptin |
|  |  | or | medication | NLM:RXNORM:10635 | tolbutamide |
|  |  | or | medication | NLM:RXNORM:10633 | tolazamide |
|  |  | or | medication | NLM:VA:HS501 | INSULIN |
|  |  | or | medication | NLM:RXNORM:60548 | exenatide |
|  |  | or | medication | NLM:RXNORM:139953 | pramlintide |
|  |  | or | medication | NLM:RXNORM:1440051 | lixisenatide |
|  |  | or | medication | NLM:RXNORM:1551291 | dulaglutide |
|  |  | or | medication | NLM:RXNORM:2621880 | teplizumab |
|  | date constraint | | The terms in this group occurred at any time | | |
| Group 2 | | | | | |
|  | **Group 2A seizure or epilepsy on an ASM** | | | | |
|  | must have | any of | diagnosis | UMLS:ICD10CM:R56 | Convulsions, not elsewhere classified |
|  |  |  | diagnosis | UMLS:ICD10CM:G40 | Epilepsy and recurrent seizures |
|  |  | and any of | medication | NLM:RXNORM:114477 | levetiracetam |
|  |  |  | medication | NLM:RXNORM:21241 | clobazam |
|  |  |  | medication | NLM:RXNORM:1356552 | perampanel |
|  |  |  | medication | NLM:RXNORM:1482502 | eslicarbazepine |
|  |  |  | medication | NLM:RXNORM:14851 | vigabatrin |
|  |  |  | medication | NLM:RXNORM:1739745 | brivaracetam |
|  |  |  | medication | NLM:RXNORM:2002 | carbamazepine |
|  |  |  | medication | NLM:RXNORM:2045371 | cannabidiol |
|  |  |  | medication | NLM:RXNORM:2054968 | stiripentol |
|  |  |  | medication | NLM:RXNORM:2265690 | cenobamate |
|  |  |  | medication | NLM:RXNORM:24812 | felbamate |
|  |  |  | medication | NLM:RXNORM:2604689 | ganaxolone |
|  |  |  | medication | NLM:RXNORM:28439 | lamotrigine |
|  |  |  | medication | NLM:RXNORM:31914 | tiagabine |
|  |  |  | medication | NLM:RXNORM:32624 | oxcarbazepine |
|  |  |  | medication | NLM:RXNORM:38404 | topiramate |
|  |  |  | medication | NLM:RXNORM:39998 | zonisamide |
|  |  |  | medication | NLM:RXNORM:40254 | valproate |
|  |  |  | medication | NLM:RXNORM:4135 | ethosuximide |
|  |  |  | medication | NLM:RXNORM:4328 | fenfluramine |
|  |  |  | medication | NLM:RXNORM:47858 | methsuximide |
|  |  |  | medication | NLM:RXNORM:623400 | lacosamide |
|  |  |  | medication | NLM:RXNORM:69036 | rufinamide |
|  |  |  | medication | NLM:RXNORM:72236 | fosphenytoin |
|  |  |  | medication | NLM:RXNORM:8183 | phenytoin |
|  |  |  | medication | NLM:RXNORM:8691 | primidone |
|  |  |  | medication | NLM:RXNORM:8134 | phenobarbital |
|  | date constraint | | The terms in this group occurred at any time | | |
|  | event relationship | | The first instance of weight loss therapy (tirzepatide/liraglutide/semaglutide/orlistat/surgery) occurred on or after the first instance of seizure or epilepsy on an ASM | | |
|  | **Group 2B weight loss therapy (tirzepatide/liraglutide/semaglutide/orlistat/surgery)** | | | | |
|  | must have | any of | medication | NLM:RXNORM:2601723 | tirzepatide |
|  |  |  | medication | NLM:RXNORM:475968 | liraglutide |
|  |  |  | medication | NLM:RXNORM:1991302 | semaglutide |
|  |  |  | medication | NLM:RXNORM:37925 | orlistat |
|  |  |  | procedure | UMLS:CPT:1007385 | Bariatric Surgery Procedures |

## Case-ascertainment accuracy

Studies validating the diagnostic accuracy of ICD-10 coding for T2DM or epilepsy in TriNetX are unavailable. However, there are studies validating the diagnostic accuracy of these codes, including in regions and settings that TriNetX sources data from.^17 18^ For T2DM, the E11 coding we used has been shown to identify T2DM with positive predictive values (PPVs) of 86.0–92.5%, sensitivities of 87.9–93.3%, negative predictive values (NPVs) of 95.1–98.0%, and specificities of 94.2–97.8%.^17^ As we showed previously, G40–41 disease coding for epilepsy–status epilepticus and/or R56.9 symptom coding for seizures, alongside co-prescribed ASMs, accurately capture epilepsy within electronic healthcare datasets.^18-21^ This spans multiple studies in a variety of healthcare settings and countries.^18-21^ This strategy is estimated to yield 80–90% sensitivity, PPV, and NPV, with specificity approaching 100%.^18-21^ The corresponding F1 scores are also high at 0.86.^21^

# **Supplement 2: Antiseizure medications considered**

• RxNorm 114477: Levetiracetam

• RxNorm 1356552: Perampanel

• RxNorm 1482502: Eslicarbazepine

• RxNorm 14851: Vigabatrin

• RxNorm 1739745: Brivaracetam

• RxNorm 2002: Carbamazepine

• RxNorm 2045371: Cannabidiol

• RxNorm 2054968: Stiripentol

• RxNorm 21241: Clobazam

• RxNorm 2265690: Cenobamate

• RxNorm 24812: Felbamate

• RxNorm 28439: Lamotrigine

• RxNorm 31914: Tiagabine

• RxNorm 32624: Oxcarbazepine

• RxNorm 32894: Paramethadione

• RxNorm 33309: Phensuximide

• RxNorm 38404: Topiramate

• RxNorm 39998: Zonisamide

• RxNorm 40254: Valproate

• RxNorm 4135: Ethosuximide

• RxNorm 4328: Fenfluramine

• RxNorm 47858: Methsuximide

• RxNorm 623400: Lacosamide

• RxNorm 69036: Rufinamide

• RxNorm 72236: Fosphenytoin

• RxNorm 8183: Phenytoin

• RxNorm 8691: Primidone

• RxNorm 8134: Phenobarbital

# Supplement 3: Coded outcome definitions

## Cohort 1 (antiepileptogenesis)

Table below outlines the definitions for each outcome and the analysis specifications.

| Incident seizure | | | | |
| --- | --- | --- | --- | --- |
|  | **Outcome definition** | | | |
|  | | Diagnosis | UMLS:ICD10CM:R56 | Convulsions, not elsewhere classified |
|  | **Settings for the performed analyses** | | | |
|  | | Kaplan - Meier survival analysis | | excluding patients with outcome prior to the time window |
| Incident epilepsy coding | | | | |
|  | **Outcome definition** | | | |
|  | | Diagnosis | UMLS:ICD10CM:G40 | Epilepsy and recurrent seizures |
|  | **Settings for the performed analyses** | | | |
|  | | Kaplan - Meier survival analysis | | excluding patients with outcome prior to the time window |
| Incident ASM prescription | | | | |
|  | **Outcome definition** | | | |
|  | | Medication | NLM:RXNORM:114477 | levetiracetam |
|  | | Medication | NLM:RXNORM:1356552 | perampanel |
|  | | Medication | NLM:RXNORM:1482502 | eslicarbazepine |
|  | | Medication | NLM:RXNORM:14851 | vigabatrin |
|  | | Medication | NLM:RXNORM:1739745 | brivaracetam |
|  | | Medication | NLM:RXNORM:2002 | carbamazepine |
|  | | Medication | NLM:RXNORM:2045371 | cannabidiol |
|  | | Medication | NLM:RXNORM:2054968 | stiripentol |
|  | | Medication | NLM:RXNORM:21241 | clobazam |
|  | | Medication | NLM:RXNORM:2265690 | cenobamate |
|  | | Medication | NLM:RXNORM:24812 | felbamate |
|  | | Medication | NLM:RXNORM:2604689 | ganaxolone |
|  | | Medication | NLM:RXNORM:28439 | lamotrigine |
|  | | Medication | NLM:RXNORM:31914 | tiagabine |
|  | | Medication | NLM:RXNORM:32624 | oxcarbazepine |
|  | | Medication | NLM:RXNORM:38404 | topiramate |
|  | | Medication | NLM:RXNORM:39998 | zonisamide |
|  | | Medication | NLM:RXNORM:40254 | valproate |
|  | | Medication | NLM:RXNORM:4135 | ethosuximide |
|  | | Medication | NLM:RXNORM:4328 | fenfluramine |
|  | | Medication | NLM:RXNORM:47858 | methsuximide |
|  | | Medication | NLM:RXNORM:623400 | lacosamide |
|  | | Medication | NLM:RXNORM:69036 | rufinamide |
|  | | Medication | NLM:RXNORM:72236 | fosphenytoin |
|  | | Medication | NLM:RXNORM:8183 | phenytoin |
|  | | Medication | NLM:RXNORM:8691 | primidone |
|  | | Medication | NLM:RXNORM:8134 | phenobarbital |
|  | **Settings for the performed analyses** | | | |
|  | | Kaplan - Meier survival analysis | | excluding patients with outcome prior to the time window |
| Death | | | | |
|  | **Outcome definition** | | | |
|  | | Demographics | Deceased | Deceased |
|  | **Settings for the performed analyses** | | | |
|  | | Kaplan - Meier survival analysis | | excluding patients with outcome prior to the time window |

## Cohort 2 (seizure control)

Table below outlines the definitions for each outcome and the analysis specifications.

| Next coded seizure | | | | |
| --- | --- | --- | --- | --- |
|  | **Outcome definition** | | | |
|  | | Diagnosis | UMLS:ICD10CM:R56 | Convulsions, not elsewhere classified |
|  | **Settings for the performed analyses** | | | |
|  | | Kaplan - Meier survival analysis | | including patients with outcome prior to the time window |
| ED attendance or hospital admission | | | | |
|  | **Outcome definition** | | | |
|  | | Visit | UMLS:HL7V3.0:VisitType:EMER | Visit: Emergency |
|  | | Visit | UMLS:HL7V3.0:VisitType:IMP | Visit: Inpatient Encounter |
|  | **Settings for the performed analyses** | | | |
|  | | Kaplan - Meier survival analysis | | including patients with outcome prior to the time window |
| Falls, injuries, burns | | | | |
|  | **Outcome definition** | | | |
|  | | Diagnosis | UMLS:ICD10CM:W00-W19 | Slipping, tripping, stumbling and falls |
|  | | Diagnosis | UMLS:ICD10CM:S00-S09 | Injuries to the head |
|  | | Diagnosis | UMLS:ICD10CM:S60-S69 | Injuries to the wrist, hand and fingers |
|  | | Diagnosis | UMLS:ICD10CM:S80-S89 | Injuries to the knee and lower leg |
|  | | Diagnosis | UMLS:ICD10CM:S90-S99 | Injuries to the ankle and foot |
|  | | Diagnosis | UMLS:ICD10CM:S30-S39 | Injuries to the abdomen, lower back, lumbar spine, pelvis and external genitals |
|  | | Diagnosis | UMLS:ICD10CM:S40-S49 | Injuries to the shoulder and upper arm |
|  | | Diagnosis | UMLS:ICD10CM:S20-S29 | Injuries to the thorax |
|  | | Diagnosis | UMLS:ICD10CM:S50-S59 | Injuries to the elbow and forearm |
|  | | Diagnosis | UMLS:ICD10CM:S10-S19 | Injuries to the neck |
|  | | Diagnosis | UMLS:ICD10CM:S70-S79 | Injuries to the hip and thigh |
|  | | Diagnosis | UMLS:ICD10CM:T07-T07 | Injuries involving multiple body regions (T07) |
|  | | Diagnosis | UMLS:ICD10CM:T14 | Injury of unspecified body region |
|  | | Diagnosis | UMLS:ICD10CM:T20-T25 | Burns and corrosions of external body surface, specified by site |
|  | | Diagnosis | UMLS:ICD10CM:T30-T32 | Burns and corrosions of multiple and unspecified body regions |
|  | **Settings for the performed analyses** | | | |
|  | | Kaplan - Meier survival analysis | | including patients with outcome prior to the time window |
| Net potentially seizure-related morbidity | | | | |
|  | **Outcome definition** | | | |
|  | | Diagnosis | UMLS:ICD10CM:R56 | Convulsions, not elsewhere classified |
|  | | Visit | UMLS:HL7V3.0:VisitType:EMER | Visit: Emergency |
|  | | Visit | UMLS:HL7V3.0:VisitType:IMP | Visit: Inpatient Encounter |
|  | | Diagnosis | UMLS:ICD10CM:W00-W19 | Slipping, tripping, stumbling and falls |
|  | | Diagnosis | UMLS:ICD10CM:S00-S09 | Injuries to the head |
|  | | Diagnosis | UMLS:ICD10CM:S60-S69 | Injuries to the wrist, hand and fingers |
|  | | Diagnosis | UMLS:ICD10CM:S80-S89 | Injuries to the knee and lower leg |
|  | | Diagnosis | UMLS:ICD10CM:S90-S99 | Injuries to the ankle and foot |
|  | | Diagnosis | UMLS:ICD10CM:S30-S39 | Injuries to the abdomen, lower back, lumbar spine, pelvis and external genitals |
|  | | Diagnosis | UMLS:ICD10CM:S40-S49 | Injuries to the shoulder and upper arm |
|  | | Diagnosis | UMLS:ICD10CM:S20-S29 | Injuries to the thorax |
|  | | Diagnosis | UMLS:ICD10CM:S50-S59 | Injuries to the elbow and forearm |
|  | | Diagnosis | UMLS:ICD10CM:S10-S19 | Injuries to the neck |
|  | | Diagnosis | UMLS:ICD10CM:S70-S79 | Injuries to the hip and thigh |
|  | | Diagnosis | UMLS:ICD10CM:T07-T07 | Injuries involving multiple body regions (T07) |
|  | | Diagnosis | UMLS:ICD10CM:T14 | Injury of unspecified body region |
|  | | Diagnosis | UMLS:ICD10CM:T20-T25 | Burns and corrosions of external body surface, specified by site |
|  | | Diagnosis | UMLS:ICD10CM:T30-T32 | Burns and corrosions of multiple and unspecified body regions |
|  | **Settings for the performed analyses** | | | |
|  | | Kaplan - Meier survival analysis | | including patients with outcome prior to the time window |
| Death | | | | |
|  | **Outcome definition** | | | |
|  | | Demographics | Deceased | Deceased |
|  | **Settings for the performed analyses** | | | |
|  | | Kaplan - Meier survival analysis | | excluding patients with outcome prior to the time window |

# Supplement 4: Propensity score matching results

## Cohort 1 (antiepileptogenesis)

Propensity score matching was performed on all listed characteristics. Characteristics of the cohorts before and after matching are summarized in the table below.

| **Cohort 1 and cohort 2 patient count before and after propensity score matching** | | | | | | | | | | | | |
| --- | --- | --- | --- | --- | --- | --- | --- | --- | --- | --- | --- | --- |
|  | | | Cohort | | | Patient count before matching | | | | Patient count after matching | | |
|  | | | 1 - Non-diabetic seizure risk factor group commenced on metformin_v16 | | | 7,325 | | | | 2,529 | | |
|  | | | 2 - Non-diabetic seizure risk factor group commenced on other WL therapies_v16 | | | 5,666 | | | | 2,529 | | |
| **Propensity score density function - Before and after matching (cohort 1 - purple, cohort 2 - green)** | | | | | | | | | | | | |
|  |  | |  | | | |  | | | | | |
| **Cohort 1 (N = 7,325) and cohort 2 (N = 5,666) characteristics before propensity score matching** | | | | | | | | | | | | |
|  | **Demographics** | | | | | | | | | | | |
|  |  | Cohort | | |  | Mean ± SD | | Patients | % of Cohort | | P-Value | Std diff. |
|  |  | 1 2 | | AI | Age at Index | 51.2 +/- 21.2 45.6 +/- 16.4 | | 7,315 5,655 | 100% 100% | | <0.001 | 0.295 |
|  |  | 1 2 | | 2106-3 | White |  | | 3,747 4,213 | 51.2% 74.5% | | <0.001 | 0.496 |
|  |  | 1 2 | | UNK | Unknown Race |  | | 2,097 493 | 28.7% 8.7% | | <0.001 | 0.529 |
|  |  | 1 2 | | F | Female |  | | 4,470 4,424 | 61.1% 78.2% | | <0.001 | 0.379 |
|  |  | 1 2 | | 2054-5 | Black or African American |  | | 791 555 | 10.8% 9.8% | | 0.064 | 0.033 |
|  |  | 1 2 | | M | Male |  | | 2,836 1,228 | 38.8% 21.7% | | <0.001 | 0.378 |
|  |  | 1 2 | | 2131-1 | Other Race |  | | 439 265 | 6.0% 4.7% | | 0.001 | 0.059 |
|  |  | 1 2 | | 2028-9 | Asian |  | | 201 92 | 2.7% 1.6% | | <0.001 | 0.077 |
|  | **Diagnosis** | | | | | | | | | | | |
|  |  | Cohort | | |  | Mean ± SD | | Patients | % of Cohort | | P-Value | Std diff. |
|  |  | 1 2 | | V00-Y99 | External causes of morbidity |  | | 1,900 2,091 | 26.0% 37.0% | | <0.001 | 0.239 |
|  |  | 1 2 | | F01-F99 | Mental, Behavioral and Neurodevelopmental disorders |  | | 2,021 3,485 | 27.6% 61.6% | | <0.001 | 0.728 |
|  |  | 1 2 | | E65-E68 | Overweight, obesity and other hyperalimentation |  | | 821 3,269 | 11.2% 57.8% | | <0.001 | 1.124 |
|  |  | 1 2 | | E28.2 | Polycystic ovarian syndrome |  | | 578 196 | 7.9% 3.5% | | <0.001 | 0.192 |
|  |  | 1 2 | | A00-B99 | Certain infectious and parasitic diseases |  | | 1,185 2,231 | 16.2% 39.5% | | <0.001 | 0.537 |
|  |  | 1 2 | | S00-S09 | Injuries to the head |  | | 5,021 4,655 | 68.6% 82.3% | | <0.001 | 0.322 |
|  |  | 1 2 | | I60-I69 | Cerebrovascular diseases |  | | 1,720 910 | 23.5% 16.1% | | <0.001 | 0.187 |
|  |  | 1 2 | | I10-I1A | Hypertensive diseases |  | | 1,014 1,581 | 13.9% 28.0% | | <0.001 | 0.352 |
|  |  | 1 2 | | J00-J99 | Diseases of the respiratory system |  | | 1,973 3,263 | 27.0% 57.7% | | <0.001 | 0.654 |
|  |  | 1 2 | | K00-K95 | Diseases of the digestive system |  | | 1,604 2,959 | 21.9% 52.3% | | <0.001 | 0.663 |
|  |  | 1 2 | | E70-E88 | Metabolic disorders |  | | 1,066 2,159 | 14.6% 38.2% | | <0.001 | 0.556 |
|  |  | 1 2 | | R56 | Convulsions, not elsewhere classified |  | | 208 170 | 2.8% 3.0% | | 0.585 | 0.010 |
|  |  | 1 2 | | G80-G83 | Cerebral palsy and other paralytic syndromes |  | | 107 117 | 1.5% 2.1% | | 0.009 | 0.046 |
|  |  | 1 2 | | G89-G99 | Other disorders of the nervous system |  | | 812 1,673 | 11.1% 29.6% | | <0.001 | 0.472 |
|  |  | 1 2 | | Z00-Z99 | Factors influencing health status and contact with health services |  | | 2,767 3,885 | 37.8% 68.7% | | <0.001 | 0.651 |
|  |  | 1 2 | | M00-M99 | Diseases of the musculoskeletal system and connective tissue |  | | 2,576 3,984 | 35.2% 70.5% | | <0.001 | 0.754 |
|  |  | 1 2 | | N00-N99 | Diseases of the genitourinary system |  | | 1,621 2,876 | 22.2% 50.9% | | <0.001 | 0.624 |
|  |  | 1 2 | | C00-D49 | Neoplasms |  | | 1,368 1,648 | 18.7% 29.1% | | <0.001 | 0.247 |
|  |  | 1 2 | | Q00-Q99 | Congenital malformations, deformations and chromosomal abnormalities |  | | 300 475 | 4.1% 8.4% | | <0.001 | 0.178 |
|  |  | 1 2 | | D50-D89 | Diseases of the blood and blood-forming organs and certain disorders involving the immune mechanism |  | | 430 1,074 | 5.9% 19.0% | | <0.001 | 0.405 |
|  |  | 1 2 | | E00-E07 | Disorders of thyroid gland |  | | 473 1,085 | 6.5% 19.2% | | <0.001 | 0.387 |
|  |  | 1 2 | | E50-E64 | Other nutritional deficiencies |  | | 388 1,237 | 5.3% 21.9% | | <0.001 | 0.498 |
|  |  | 1 2 | | S90-S99 | Injuries to the ankle and foot |  | | 536 740 | 7.3% 13.1% | | <0.001 | 0.191 |
|  |  | 1 2 | | S80-S89 | Injuries to the knee and lower leg |  | | 654 888 | 8.9% 15.7% | | <0.001 | 0.207 |
|  |  | 1 2 | | S60-S69 | Injuries to the wrist, hand and fingers |  | | 694 757 | 9.5% 13.4% | | <0.001 | 0.123 |
|  |  | 1 2 | | S40-S49 | Injuries to the shoulder and upper arm |  | | 494 585 | 6.8% 10.3% | | <0.001 | 0.129 |
|  |  | 1 2 | | T80-T88 | Complications of surgical and medical care, not elsewhere classified |  | | 141 313 | 1.9% 5.5% | | <0.001 | 0.191 |
|  |  | 1 2 | | S30-S39 | Injuries to the abdomen, lower back, lumbar spine, pelvis and external genitals |  | | 495 577 | 6.8% 10.2% | | <0.001 | 0.124 |
|  |  | 1 2 | | S10-S19 | Injuries to the neck |  | | 748 700 | 10.2% 12.4% | | <0.001 | 0.068 |
|  |  | 1 2 | | T66-T78 | Other and unspecified effects of external causes |  | | 173 298 | 2.4% 5.3% | | <0.001 | 0.152 |
|  |  | 1 2 | | T14-T14 | Injury of unspecified body region (T14) |  | | 278 375 | 3.8% 6.6% | | <0.001 | 0.128 |
|  |  | 1 2 | | I30-I5A | Other forms of heart disease |  | | 493 857 | 6.7% 15.2% | | <0.001 | 0.272 |
|  |  | 1 2 | | I80-I89 | Diseases of veins, lymphatic vessels and lymph nodes, not elsewhere classified |  | | 145 377 | 2.0% 6.7% | | <0.001 | 0.232 |
|  |  | 1 2 | | I70-I79 | Diseases of arteries, arterioles and capillaries |  | | 174 321 | 2.4% 5.7% | | <0.001 | 0.168 |
|  |  | 1 2 | | H60-H95 | Diseases of the ear and mastoid process |  | | 829 1,408 | 11.3% 24.9% | | <0.001 | 0.358 |
|  |  | 1 2 | | U00-U85 | Codes for special purposes |  | | 372 834 | 5.1% 14.7% | | <0.001 | 0.328 |
|  |  | 1 2 | | G47 | Sleep disorders |  | | 594 1,570 | 8.1% 27.8% | | <0.001 | 0.530 |
|  |  | 1 2 | | G43 | Migraine |  | | 468 1,099 | 6.4% 19.4% | | <0.001 | 0.396 |
|  |  | 1 2 | | G44 | Other headache syndromes |  | | 431 742 | 5.9% 13.1% | | <0.001 | 0.248 |
|  |  | 1 2 | | R51 | Headache |  | | 908 1,389 | 12.4% 24.6% | | <0.001 | 0.317 |
|  |  | 1 2 | | R55 | Syncope and collapse |  | | 269 452 | 3.7% 8.0% | | <0.001 | 0.185 |
|  |  | 1 2 | | R53 | Malaise and fatigue |  | | 454 1,039 | 6.2% 18.4% | | <0.001 | 0.377 |
|  |  | 1 2 | | R00-R09 | Symptoms and signs involving the circulatory and respiratory systems |  | | 1,583 3,064 | 21.6% 54.2% | | <0.001 | 0.712 |
|  |  | 1 2 | | R25-R29 | Symptoms and signs involving the nervous and musculoskeletal systems |  | | 470 777 | 6.4% 13.7% | | <0.001 | 0.245 |
|  |  | 1 2 | | R10-R19 | Symptoms and signs involving the digestive system and abdomen |  | | 1,287 2,436 | 17.6% 43.1% | | <0.001 | 0.577 |
|  | **Procedure** | | | | | | | | | | | |
|  |  | Cohort | | |  | Mean ± SD | | Patients | % of Cohort | | P-Value | Std diff. |
|  |  | 1 2 | | 1010251 | Radiology Procedures |  | | 2,813 3,596 | 38.5% 63.6% | | <0.001 | 0.519 |
|  |  | 1 2 | | 1011136 | Pathology and Laboratory Procedures |  | | 1,706 3,253 | 23.3% 57.5% | | <0.001 | 0.744 |
|  |  | 1 2 | | 1012569 | Medicine Services and Procedures |  | | 2,383 3,442 | 32.6% 60.9% | | <0.001 | 0.591 |
|  |  | 1 2 | | 1013625 | Evaluation and Management |  | | 3,486 3,516 | 47.7% 62.2% | | <0.001 | 0.295 |
|  |  | 1 2 | | 1003143 | Surgery |  | | 1,878 3,327 | 25.7% 58.8% | | <0.001 | 0.713 |
|  | **Medication** | | | | | | | | | | | |
|  |  | Cohort | | |  | Mean ± SD | | Patients | % of Cohort | | P-Value | Std diff. |
|  |  | 1 2 | | CN600 | ANTIDEPRESSANTS |  | | 2,567 3,005 | 35.1% 53.1% | | <0.001 | 0.370 |
|  |  | 1 2 | | CN300 | SEDATIVES/HYPONTICS |  | | 1,933 2,544 | 26.4% 45.0% | | <0.001 | 0.395 |
|  |  | 1 2 | | CN700 | ANTIPSYCHOTICS |  | | 915 558 | 12.5% 9.9% | | <0.001 | 0.084 |
|  |  | 1 2 | | CN800 | CNS STIMULANTS |  | | 588 876 | 8.0% 15.5% | | <0.001 | 0.233 |
|  |  | 1 2 | | CV000 | CARDIOVASCULAR MEDICATIONS |  | | 5,269 3,895 | 72.0% 68.9% | | <0.001 | 0.069 |
|  |  | 1 2 | | 25480 | gabapentin |  | | 754 840 | 10.3% 14.9% | | <0.001 | 0.137 |
|  |  | 1 2 | | CN101 | OPIOID ANALGESICS |  | | 2,272 2,991 | 31.1% 52.9% | | <0.001 | 0.454 |
|  |  | 1 2 | | CN302 | BENZODIAZEPINE DERIVATIVE SEDATIVES/HYPNOTICS |  | | 1,666 2,227 | 22.8% 39.4% | | <0.001 | 0.365 |
|  |  | 1 2 | | CN309 | SEDATIVES/HYPNOTICS,OTHER |  | | 566 826 | 7.7% 14.6% | | <0.001 | 0.219 |
|  |  | 1 2 | | AM000 | ANTIMICROBIALS |  | | 3,458 4,285 | 47.3% 75.8% | | <0.001 | 0.613 |
|  |  | 1 2 | | AN000 | ANTINEOPLASTICS |  | | 449 505 | 6.1% 8.9% | | <0.001 | 0.106 |
|  |  | 1 2 | | 187832 | pregabalin |  | | 206 244 | 2.8% 4.3% | | <0.001 | 0.081 |
|  |  | 1 2 | | RE000 | RESPIRATORY TRACT MEDICATIONS |  | | 3,265 3,959 | 44.6% 70.0% | | <0.001 | 0.531 |
|  |  | 1 2 | | CN200 | ANESTHETICS |  | | 1,248 2,232 | 17.1% 39.5% | | <0.001 | 0.514 |
|  |  | 1 2 | | CN550 | ANTIVERTIGO AGENTS |  | | 307 589 | 4.2% 10.4% | | <0.001 | 0.241 |
|  |  | 1 2 | | IM000 | IMMUNOLOGICAL AGENTS |  | | 1,460 2,914 | 20.0% 51.5% | | <0.001 | 0.698 |
|  |  | 1 2 | | AU000 | AUTONOMIC MEDICATIONS |  | | 1,383 1,847 | 18.9% 32.7% | | <0.001 | 0.318 |
|  | **Laboratory** | | | | | | | | | | | |
|  |  | Cohort | | |  | Mean ± SD | | Patients | % of Cohort | | P-Value | Std diff. |
|  |  | 1 2 | | 9083 | BMI | 31.4 +/- 7.5 35.2 +/- 7.3 | | 3,584 3,955 | 49.0% 69.9% | | <0.001 | 0.517 |
|  |  | 1 2 | |  | 0 - 0 kg/m2 |  | | 3,592 3,958 | 49.1% 70.0% | | <0.001 | 0.436 |
|  |  | 1 2 | | 9025 | Glucose [Mass/volume] in Serum, Plasma or Blood | 87.1 +/- 8.7 87.2 +/- 7.8 | | 1,296 3,280 | 17.7% 58.0% | | 0.745 | 0.010 |
|  |  | 1 2 | |  | 0 - 0 mg/dL |  | | 1,296 3,280 | 17.7% 58.0% | | <0.001 | 0.913 |
|  |  | 1 2 | | 9037 | Hemoglobin A1c/Hemoglobin.total in Blood | 5.2 +/- 0.5 5.2 +/- 0.3 | | 702 2,077 | 9.6% 36.7% | | 0.006 | 0.103 |
|  |  | 1 2 | |  | 0 - 0 % |  | | 702 2,077 | 9.6% 36.7% | | <0.001 | 0.679 |
| **Cohort 1 (N = 2,529) and cohort 2 (N = 2,529) characteristics after propensity score matching** | | | | | | | | | | | | |
|  | **Demographics** | | | | | | | | | | | |
|  |  | Cohort | | |  | Mean ± SD | | Patients | % of Cohort | | P-Value | Std diff. |
|  |  | 1 2 | | AI | Age at Index | 45.4 +/- 21.4 46.7 +/- 17.1 | | 2,529 2,529 | 100% 100% | | 0.011 | 0.071 |
|  |  | 1 2 | | 2106-3 | White |  | | 1,702 1,695 | 67.3% 67.0% | | 0.834 | 0.006 |
|  |  | 1 2 | | UNK | Unknown Race |  | | 364 382 | 14.4% 15.1% | | 0.475 | 0.020 |
|  |  | 1 2 | | F | Female |  | | 1,842 1,840 | 72.8% 72.8% | | 0.950 | 0.002 |
|  |  | 1 2 | | 2054-5 | Black or African American |  | | 254 234 | 10.0% 9.3% | | 0.341 | 0.027 |
|  |  | 1 2 | | M | Male |  | | 685 687 | 27.1% 27.2% | | 0.950 | 0.002 |
|  |  | 1 2 | | 2131-1 | Other Race |  | | 127 140 | 5.0% 5.5% | | 0.414 | 0.023 |
|  |  | 1 2 | | 2028-9 | Asian |  | | 64 62 | 2.5% 2.5% | | 0.857 | 0.005 |
|  | **Diagnosis** | | | | | | | | | | | |
|  |  | Cohort | | |  | Mean ± SD | | Patients | % of Cohort | | P-Value | Std diff. |
|  |  | 1 2 | | V00-Y99 | External causes of morbidity |  | | 835 818 | 33.0% 32.3% | | 0.610 | 0.014 |
|  |  | 1 2 | | F01-F99 | Mental, Behavioral and Neurodevelopmental disorders |  | | 1,191 1,165 | 47.1% 46.1% | | 0.464 | 0.021 |
|  |  | 1 2 | | E65-E68 | Overweight, obesity and other hyperalimentation |  | | 715 734 | 28.3% 29.0% | | 0.555 | 0.017 |
|  |  | 1 2 | | E28.2 | Polycystic ovarian syndrome |  | | 174 162 | 6.9% 6.4% | | 0.498 | 0.019 |
|  |  | 1 2 | | A00-B99 | Certain infectious and parasitic diseases |  | | 694 687 | 27.4% 27.2% | | 0.825 | 0.006 |
|  |  | 1 2 | | S00-S09 | Injuries to the head |  | | 1,974 1,967 | 78.1% 77.8% | | 0.812 | 0.007 |
|  |  | 1 2 | | I60-I69 | Cerebrovascular diseases |  | | 466 473 | 18.4% 18.7% | | 0.800 | 0.007 |
|  |  | 1 2 | | I10-I1A | Hypertensive diseases |  | | 562 566 | 22.2% 22.4% | | 0.893 | 0.004 |
|  |  | 1 2 | | J00-J99 | Diseases of the respiratory system |  | | 1,123 1,117 | 44.4% 44.2% | | 0.865 | 0.005 |
|  |  | 1 2 | | K00-K95 | Diseases of the digestive system |  | | 986 981 | 39.0% 38.8% | | 0.885 | 0.004 |
|  |  | 1 2 | | E70-E88 | Metabolic disorders |  | | 706 722 | 27.9% 28.5% | | 0.617 | 0.014 |
|  |  | 1 2 | | R56 | Convulsions, not elsewhere classified |  | | 90 88 | 3.6% 3.5% | | 0.879 | 0.004 |
|  |  | 1 2 | | G80-G83 | Cerebral palsy and other paralytic syndromes |  | | 55 53 | 2.2% 2.1% | | 0.846 | 0.005 |
|  |  | 1 2 | | G89-G99 | Other disorders of the nervous system |  | | 506 503 | 20.0% 19.9% | | 0.916 | 0.003 |
|  |  | 1 2 | | Z00-Z99 | Factors influencing health status and contact with health services |  | | 1,482 1,456 | 58.6% 57.6% | | 0.459 | 0.021 |
|  |  | 1 2 | | M00-M99 | Diseases of the musculoskeletal system and connective tissue |  | | 1,442 1,435 | 57.0% 56.7% | | 0.842 | 0.006 |
|  |  | 1 2 | | N00-N99 | Diseases of the genitourinary system |  | | 967 980 | 38.2% 38.8% | | 0.707 | 0.011 |
|  |  | 1 2 | | C00-D49 | Neoplasms |  | | 573 589 | 22.7% 23.3% | | 0.593 | 0.015 |
|  |  | 1 2 | | Q00-Q99 | Congenital malformations, deformations and chromosomal abnormalities |  | | 182 186 | 7.2% 7.4% | | 0.829 | 0.006 |
|  |  | 1 2 | | D50-D89 | Diseases of the blood and blood-forming organs and certain disorders involving the immune mechanism |  | | 305 330 | 12.1% 13.0% | | 0.289 | 0.030 |
|  |  | 1 2 | | E00-E07 | Disorders of thyroid gland |  | | 332 321 | 13.1% 12.7% | | 0.645 | 0.013 |
|  |  | 1 2 | | E50-E64 | Other nutritional deficiencies |  | | 298 307 | 11.8% 12.1% | | 0.697 | 0.011 |
|  |  | 1 2 | | S90-S99 | Injuries to the ankle and foot |  | | 268 284 | 10.6% 11.2% | | 0.471 | 0.020 |
|  |  | 1 2 | | S80-S89 | Injuries to the knee and lower leg |  | | 297 310 | 11.7% 12.3% | | 0.574 | 0.016 |
|  |  | 1 2 | | S60-S69 | Injuries to the wrist, hand and fingers |  | | 299 293 | 11.8% 11.6% | | 0.793 | 0.007 |
|  |  | 1 2 | | S40-S49 | Injuries to the shoulder and upper arm |  | | 211 208 | 8.3% 8.2% | | 0.878 | 0.004 |
|  |  | 1 2 | | T80-T88 | Complications of surgical and medical care, not elsewhere classified |  | | 99 100 | 3.9% 4.0% | | 0.942 | 0.002 |
|  |  | 1 2 | | S30-S39 | Injuries to the abdomen, lower back, lumbar spine, pelvis and external genitals |  | | 222 203 | 8.8% 8.0% | | 0.336 | 0.027 |
|  |  | 1 2 | | S10-S19 | Injuries to the neck |  | | 284 274 | 11.2% 10.8% | | 0.654 | 0.013 |
|  |  | 1 2 | | T66-T78 | Other and unspecified effects of external causes |  | | 97 98 | 3.8% 3.9% | | 0.942 | 0.002 |
|  |  | 1 2 | | T14-T14 | Injury of unspecified body region (T14) |  | | 136 111 | 5.4% 4.4% | | 0.103 | 0.046 |
|  |  | 1 2 | | I30-I5A | Other forms of heart disease |  | | 302 311 | 11.9% 12.3% | | 0.698 | 0.011 |
|  |  | 1 2 | | I80-I89 | Diseases of veins, lymphatic vessels and lymph nodes, not elsewhere classified |  | | 101 105 | 4.0% 4.2% | | 0.776 | 0.008 |
|  |  | 1 2 | | I70-I79 | Diseases of arteries, arterioles and capillaries |  | | 98 109 | 3.9% 4.3% | | 0.435 | 0.022 |
|  |  | 1 2 | | H60-H95 | Diseases of the ear and mastoid process |  | | 458 447 | 18.1% 17.7% | | 0.687 | 0.011 |
|  |  | 1 2 | | U00-U85 | Codes for special purposes |  | | 220 214 | 8.7% 8.5% | | 0.763 | 0.008 |
|  |  | 1 2 | | G47 | Sleep disorders |  | | 424 432 | 16.8% 17.1% | | 0.764 | 0.008 |
|  |  | 1 2 | | G43 | Migraine |  | | 317 334 | 12.5% 13.2% | | 0.475 | 0.020 |
|  |  | 1 2 | | G44 | Other headache syndromes |  | | 241 238 | 9.5% 9.4% | | 0.885 | 0.004 |
|  |  | 1 2 | | R51 | Headache |  | | 489 502 | 19.3% 19.8% | | 0.645 | 0.013 |
|  |  | 1 2 | | R55 | Syncope and collapse |  | | 176 163 | 7.0% 6.4% | | 0.465 | 0.021 |
|  |  | 1 2 | | R53 | Malaise and fatigue |  | | 285 294 | 11.3% 11.6% | | 0.691 | 0.011 |
|  |  | 1 2 | | R00-R09 | Symptoms and signs involving the circulatory and respiratory systems |  | | 979 968 | 38.7% 38.3% | | 0.751 | 0.009 |
|  |  | 1 2 | | R25-R29 | Symptoms and signs involving the nervous and musculoskeletal systems |  | | 251 266 | 9.9% 10.5% | | 0.486 | 0.020 |
|  |  | 1 2 | | R10-R19 | Symptoms and signs involving the digestive system and abdomen |  | | 809 796 | 32.0% 31.5% | | 0.695 | 0.011 |
|  | **Procedure** | | | | | | | | | | | |
|  |  | Cohort | | |  | Mean ± SD | | Patients | % of Cohort | | P-Value | Std diff. |
|  |  | 1 2 | | 1010251 | Radiology Procedures |  | | 1,355 1,302 | 53.6% 51.5% | | 0.136 | 0.042 |
|  |  | 1 2 | | 1011136 | Pathology and Laboratory Procedures |  | | 1,087 1,043 | 43.0% 41.2% | | 0.210 | 0.035 |
|  |  | 1 2 | | 1012569 | Medicine Services and Procedures |  | | 1,235 1,182 | 48.8% 46.7% | | 0.136 | 0.042 |
|  |  | 1 2 | | 1013625 | Evaluation and Management |  | | 1,313 1,293 | 51.9% 51.1% | | 0.574 | 0.016 |
|  |  | 1 2 | | 1003143 | Surgery |  | | 1,112 1,062 | 44.0% 42.0% | | 0.156 | 0.040 |
|  | **Medication** | | | | | | | | | | | |
|  |  | Cohort | | |  | Mean ± SD | | Patients | % of Cohort | | P-Value | Std diff. |
|  |  | 1 2 | | CN600 | ANTIDEPRESSANTS |  | | 1,162 1,168 | 45.9% 46.2% | | 0.866 | 0.005 |
|  |  | 1 2 | | CN300 | SEDATIVES/HYPONTICS |  | | 931 943 | 36.8% 37.3% | | 0.727 | 0.010 |
|  |  | 1 2 | | CN700 | ANTIPSYCHOTICS |  | | 328 315 | 13.0% 12.5% | | 0.583 | 0.015 |
|  |  | 1 2 | | CN800 | CNS STIMULANTS |  | | 352 331 | 13.9% 13.1% | | 0.388 | 0.024 |
|  |  | 1 2 | | CV000 | CARDIOVASCULAR MEDICATIONS |  | | 1,672 1,689 | 66.1% 66.8% | | 0.613 | 0.014 |
|  |  | 1 2 | | 25480 | gabapentin |  | | 335 330 | 13.2% 13.0% | | 0.835 | 0.006 |
|  |  | 1 2 | | CN101 | OPIOID ANALGESICS |  | | 1,140 1,123 | 45.1% 44.4% | | 0.631 | 0.014 |
|  |  | 1 2 | | CN302 | BENZODIAZEPINE DERIVATIVE SEDATIVES/HYPNOTICS |  | | 810 822 | 32.0% 32.5% | | 0.718 | 0.010 |
|  |  | 1 2 | | CN309 | SEDATIVES/HYPNOTICS,OTHER |  | | 306 312 | 12.1% 12.3% | | 0.797 | 0.007 |
|  |  | 1 2 | | AM000 | ANTIMICROBIALS |  | | 1,669 1,641 | 66.0% 64.9% | | 0.408 | 0.023 |
|  |  | 1 2 | | AN000 | ANTINEOPLASTICS |  | | 237 233 | 9.4% 9.2% | | 0.846 | 0.005 |
|  |  | 1 2 | | 187832 | pregabalin |  | | 112 105 | 4.4% 4.2% | | 0.627 | 0.014 |
|  |  | 1 2 | | RE000 | RESPIRATORY TRACT MEDICATIONS |  | | 1,524 1,516 | 60.3% 59.9% | | 0.818 | 0.006 |
|  |  | 1 2 | | CN200 | ANESTHETICS |  | | 703 676 | 27.8% 26.7% | | 0.394 | 0.024 |
|  |  | 1 2 | | CN550 | ANTIVERTIGO AGENTS |  | | 170 179 | 6.7% 7.1% | | 0.618 | 0.014 |
|  |  | 1 2 | | IM000 | IMMUNOLOGICAL AGENTS |  | | 910 900 | 36.0% 35.6% | | 0.769 | 0.008 |
|  |  | 1 2 | | AU000 | AUTONOMIC MEDICATIONS |  | | 656 633 | 25.9% 25.0% | | 0.458 | 0.021 |
|  | **Laboratory** | | | | | | | | | | | |
|  |  | Cohort | | |  | Mean ± SD | | Patients | % of Cohort | | P-Value | Std diff. |
|  |  | 1 2 | | 9083 | BMI | 32.2 +/- 8.2 34.0 +/- 7.1 | | 1,536 1,490 | 60.7% 58.9% | | <0.001 | 0.244 |
|  |  | 1 2 | |  | 0 - 0 kg/m2 |  | | 1,537 1,491 | 60.8% 59.0% | | 0.187 | 0.037 |
|  |  | 1 2 | | 9025 | Glucose [Mass/volume] in Serum, Plasma or Blood | 87.2 +/- 8.1 87.6 +/- 7.8 | | 928 909 | 36.7% 35.9% | | 0.279 | 0.051 |
|  |  | 1 2 | |  | 0 - 0 mg/dL |  | | 928 909 | 36.7% 35.9% | | 0.579 | 0.016 |
|  |  | 1 2 | | 9037 | Hemoglobin A1c/Hemoglobin.total in Blood | 5.1 +/- 0.5 5.2 +/- 0.3 | | 509 523 | 20.1% 20.7% | | 0.144 | 0.091 |
|  |  | 1 2 | |  | 0 - 0 % |  | | 509 523 | 20.1% 20.7% | | 0.625 | 0.014 |

## Cohort 2 (seizure control)

Propensity score matching was performed on all listed characteristics. Characteristics of the cohorts before and after matching are summarized in the table below.

| **Cohort 1 and cohort 2 patient count before and after propensity score matching** | | | | | | | | | | | | |
| --- | --- | --- | --- | --- | --- | --- | --- | --- | --- | --- | --- | --- |
|  | | | Cohort | | | Patient count before matching | | | | Patient count after matching | | |
|  | | | 1 - Non-diabetic PWE on an ASM commenced on metformin add-on_v13 | | | 2,085 | | | | 847 | | |
|  | | | 2 - Non-diabetic PWE on an ASM commenced on other add-on WL therapy_v13 | | | 1,619 | | | | 847 | | |
| **Propensity score density function - Before and after matching (cohort 1 - purple, cohort 2 - green)** | | | | | | | | | | | | |
|  |  | |  | | | |  | | | | | |
| **Cohort 1 (N = 2,085) and cohort 2 (N = 1,619) characteristics before propensity score matching** | | | | | | | | | | | | |
|  | **Demographics** | | | | | | | | | | | |
|  |  | Cohort | | |  | Mean ± SD | | Patients | % of Cohort | | P-Value | Std diff. |
|  |  | 1 2 | | AI | Age at Index | 40.0 +/- 19.5 42.2 +/- 15.8 | | 2,080 1,609 | 100% 100% | | <0.001 | 0.123 |
|  |  | 1 2 | | 2106-3 | White |  | | 1,204 1,184 | 57.9% 73.6% | | <0.001 | 0.335 |
|  |  | 1 2 | | UNK | Unknown Race |  | | 510 173 | 24.5% 10.8% | | <0.001 | 0.367 |
|  |  | 1 2 | | F | Female |  | | 1,353 1,230 | 65.0% 76.4% | | <0.001 | 0.253 |
|  |  | 1 2 | | 2054-5 | Black or African American |  | | 241 160 | 11.6% 9.9% | | 0.112 | 0.053 |
|  |  | 1 2 | | M | Male |  | | 725 378 | 34.9% 23.5% | | <0.001 | 0.252 |
|  |  | 1 2 | | 2131-1 | Other Race |  | | 84 55 | 4.0% 3.4% | | 0.327 | 0.033 |
|  |  | 1 2 | | 2028-9 | Asian |  | | 29 19 | 1.4% 1.2% | | 0.571 | 0.019 |
|  | **Diagnosis** | | | | | | | | | | | |
|  |  | Cohort | | |  | Mean ± SD | | Patients | % of Cohort | | P-Value | Std diff. |
|  |  | 1 2 | | V00-Y99 | External causes of morbidity |  | | 224 371 | 10.8% 23.1% | | <0.001 | 0.332 |
|  |  | 1 2 | | F01-F99 | Mental, Behavioral and Neurodevelopmental disorders |  | | 1,108 1,069 | 53.3% 66.4% | | <0.001 | 0.271 |
|  |  | 1 2 | | E65-E68 | Overweight, obesity and other hyperalimentation |  | | 394 908 | 18.9% 56.4% | | <0.001 | 0.839 |
|  |  | 1 2 | | E28.2 | Polycystic ovarian syndrome |  | | 165 61 | 7.9% 3.8% | | <0.001 | 0.177 |
|  |  | 1 2 | | A00-B99 | Certain infectious and parasitic diseases |  | | 317 521 | 15.2% 32.4% | | <0.001 | 0.411 |
|  |  | 1 2 | | S00-S09 | Injuries to the head |  | | 189 234 | 9.1% 14.5% | | <0.001 | 0.170 |
|  |  | 1 2 | | I60-I69 | Cerebrovascular diseases |  | | 165 150 | 7.9% 9.3% | | 0.134 | 0.050 |
|  |  | 1 2 | | I10-I1A | Hypertensive diseases |  | | 291 395 | 14.0% 24.5% | | <0.001 | 0.270 |
|  |  | 1 2 | | J00-J99 | Diseases of the respiratory system |  | | 539 780 | 25.9% 48.5% | | <0.001 | 0.480 |
|  |  | 1 2 | | K00-K95 | Diseases of the digestive system |  | | 602 784 | 28.9% 48.7% | | <0.001 | 0.415 |
|  |  | 1 2 | | E70-E88 | Metabolic disorders |  | | 344 545 | 16.5% 33.9% | | <0.001 | 0.407 |
|  |  | 1 2 | | G40.9 | Epilepsy, unspecified |  | | 1,019 897 | 49.0% 55.7% | | <0.001 | 0.136 |
|  |  | 1 2 | | G40.2 | Localization-related (focal) (partial) symptomatic epilepsy and epileptic syndromes with complex partial seizures |  | | 345 218 | 16.6% 13.5% | | 0.011 | 0.085 |
|  |  | 1 2 | | G40.3 | Generalized idiopathic epilepsy and epileptic syndromes |  | | 293 211 | 14.1% 13.1% | | 0.394 | 0.028 |
|  |  | 1 2 | | G40.1 | Localization-related (focal) (partial) symptomatic epilepsy and epileptic syndromes with simple partial seizures |  | | 218 206 | 10.5% 12.8% | | 0.028 | 0.072 |
|  |  | 1 2 | | G40.4 | Other generalized epilepsy and epileptic syndromes |  | | 203 127 | 9.8% 7.9% | | 0.049 | 0.066 |
|  |  | 1 2 | | G40.8 | Other epilepsy and recurrent seizures |  | | 124 112 | 6.0% 7.0% | | 0.219 | 0.041 |
|  |  | 1 2 | | R56 | Convulsions, not elsewhere classified |  | | 1,244 1,011 | 59.8% 62.8% | | 0.061 | 0.062 |
|  |  | 1 2 | | G80-G83 | Cerebral palsy and other paralytic syndromes |  | | 101 85 | 4.9% 5.3% | | 0.557 | 0.019 |
|  |  | 1 2 | | G89-G99 | Other disorders of the nervous system |  | | 367 467 | 17.6% 29.0% | | <0.001 | 0.272 |
|  |  | 1 2 | | Z00-Z99 | Factors influencing health status and contact with health services |  | | 1,076 1,181 | 51.7% 73.4% | | <0.001 | 0.459 |
|  |  | 1 2 | | M00-M99 | Diseases of the musculoskeletal system and connective tissue |  | | 694 939 | 33.4% 58.4% | | <0.001 | 0.518 |
|  |  | 1 2 | | N00-N99 | Diseases of the genitourinary system |  | | 526 747 | 25.3% 46.4% | | <0.001 | 0.452 |
|  |  | 1 2 | | C00-D49 | Neoplasms |  | | 272 352 | 13.1% 21.9% | | <0.001 | 0.233 |
|  |  | 1 2 | | Q00-Q99 | Congenital malformations, deformations and chromosomal abnormalities |  | | 214 193 | 10.3% 12.0% | | 0.101 | 0.054 |
|  |  | 1 2 | | D50-D89 | Diseases of the blood and blood-forming organs and certain disorders involving the immune mechanism |  | | 150 327 | 7.2% 20.3% | | <0.001 | 0.388 |
|  |  | 1 2 | | E00-E07 | Disorders of thyroid gland |  | | 189 281 | 9.1% 17.5% | | <0.001 | 0.249 |
|  |  | 1 2 | | E50-E64 | Other nutritional deficiencies |  | | 159 325 | 7.6% 20.2% | | <0.001 | 0.369 |
|  |  | 1 2 | | S90-S99 | Injuries to the ankle and foot |  | | 85 143 | 4.1% 8.9% | | <0.001 | 0.196 |
|  |  | 1 2 | | S80-S89 | Injuries to the knee and lower leg |  | | 82 157 | 3.9% 9.8% | | <0.001 | 0.232 |
|  |  | 1 2 | | S60-S69 | Injuries to the wrist, hand and fingers |  | | 79 115 | 3.8% 7.1% | | <0.001 | 0.148 |
|  |  | 1 2 | | S40-S49 | Injuries to the shoulder and upper arm |  | | 58 92 | 2.8% 5.7% | | <0.001 | 0.146 |
|  |  | 1 2 | | T80-T88 | Complications of surgical and medical care, not elsewhere classified |  | | 58 111 | 2.8% 6.9% | | <0.001 | 0.192 |
|  |  | 1 2 | | S30-S39 | Injuries to the abdomen, lower back, lumbar spine, pelvis and external genitals |  | | 48 83 | 2.3% 5.2% | | <0.001 | 0.151 |
|  |  | 1 2 | | S10-S19 | Injuries to the neck |  | | 46 73 | 2.2% 4.5% | | <0.001 | 0.129 |
|  |  | 1 2 | | T66-T78 | Other and unspecified effects of external causes |  | | 58 105 | 2.8% 6.5% | | <0.001 | 0.178 |
|  |  | 1 2 | | T14-T14 | Injury of unspecified body region (T14) |  | | 52 62 | 2.5% 3.9% | | 0.018 | 0.077 |
|  |  | 1 2 | | I30-I5A | Other forms of heart disease |  | | 153 218 | 7.4% 13.5% | | <0.001 | 0.203 |
|  |  | 1 2 | | I80-I89 | Diseases of veins, lymphatic vessels and lymph nodes, not elsewhere classified |  | | 50 93 | 2.4% 5.8% | | <0.001 | 0.171 |
|  |  | 1 2 | | I70-I79 | Diseases of arteries, arterioles and capillaries |  | | 42 80 | 2.0% 5.0% | | <0.001 | 0.161 |
|  |  | 1 2 | | H60-H95 | Diseases of the ear and mastoid process |  | | 241 296 | 11.6% 18.4% | | <0.001 | 0.192 |
|  |  | 1 2 | | U00-U85 | Codes for special purposes |  | | 58 147 | 2.8% 9.1% | | <0.001 | 0.271 |
|  |  | 1 2 | | G47 | Sleep disorders |  | | 364 463 | 17.5% 28.8% | | <0.001 | 0.270 |
|  |  | 1 2 | | G43 | Migraine |  | | 311 426 | 15.0% 26.5% | | <0.001 | 0.287 |
|  |  | 1 2 | | G44 | Other headache syndromes |  | | 159 224 | 7.6% 13.9% | | <0.001 | 0.203 |
|  |  | 1 2 | | R51 | Headache |  | | 320 395 | 15.4% 24.5% | | <0.001 | 0.231 |
|  |  | 1 2 | | R55 | Syncope and collapse |  | | 141 198 | 6.8% 12.3% | | <0.001 | 0.189 |
|  |  | 1 2 | | R53 | Malaise and fatigue |  | | 160 304 | 7.7% 18.9% | | <0.001 | 0.335 |
|  |  | 1 2 | | R00-R09 | Symptoms and signs involving the circulatory and respiratory systems |  | | 465 716 | 22.4% 44.5% | | <0.001 | 0.483 |
|  |  | 1 2 | | R25-R29 | Symptoms and signs involving the nervous and musculoskeletal systems |  | | 260 317 | 12.5% 19.7% | | <0.001 | 0.197 |
|  |  | 1 2 | | R10-R19 | Symptoms and signs involving the digestive system and abdomen |  | | 437 601 | 21.0% 37.4% | | <0.001 | 0.365 |
|  | **Procedure** | | | | | | | | | | | |
|  |  | Cohort | | |  | Mean ± SD | | Patients | % of Cohort | | P-Value | Std diff. |
|  |  | 1 2 | | 1010251 | Radiology Procedures |  | | 771 946 | 37.1% 58.8% | | <0.001 | 0.446 |
|  |  | 1 2 | | 1011136 | Pathology and Laboratory Procedures |  | | 660 897 | 31.7% 55.7% | | <0.001 | 0.499 |
|  |  | 1 2 | | 1012569 | Medicine Services and Procedures |  | | 940 996 | 45.2% 61.9% | | <0.001 | 0.340 |
|  |  | 1 2 | | 1013625 | Evaluation and Management |  | | 1,254 982 | 60.3% 61.0% | | 0.647 | 0.015 |
|  |  | 1 2 | | 1003143 | Surgery |  | | 584 881 | 28.1% 54.8% | | <0.001 | 0.563 |
|  | **Medication** | | | | | | | | | | | |
|  |  | Cohort | | |  | Mean ± SD | | Patients | % of Cohort | | P-Value | Std diff. |
|  |  | 1 2 | | CN600 | ANTIDEPRESSANTS |  | | 1,123 956 | 54.0% 59.4% | | 0.001 | 0.110 |
|  |  | 1 2 | | CN300 | SEDATIVES/HYPONTICS |  | | 1,101 982 | 52.9% 61.0% | | <0.001 | 0.164 |
|  |  | 1 2 | | CN700 | ANTIPSYCHOTICS |  | | 728 304 | 35% 18.9% | | <0.001 | 0.369 |
|  |  | 1 2 | | CN800 | CNS STIMULANTS |  | | 301 332 | 14.5% 20.6% | | <0.001 | 0.163 |
|  |  | 1 2 | | CV000 | CARDIOVASCULAR MEDICATIONS |  | | 1,458 1,114 | 70.1% 69.2% | | 0.573 | 0.019 |
|  |  | 1 2 | | 114477 | levetiracetam |  | | 802 618 | 38.6% 38.4% | | 0.927 | 0.003 |
|  |  | 1 2 | | 28439 | lamotrigine |  | | 616 464 | 29.6% 28.8% | | 0.607 | 0.017 |
|  |  | 1 2 | | 38404 | topiramate |  | | 480 503 | 23.1% 31.3% | | <0.001 | 0.185 |
|  |  | 1 2 | | 40254 | valproate |  | | 436 204 | 21.0% 12.7% | | <0.001 | 0.223 |
|  |  | 1 2 | | 25480 | gabapentin |  | | 299 325 | 14.4% 20.2% | | <0.001 | 0.154 |
|  |  | 1 2 | | 32624 | oxcarbazepine |  | | 207 158 | 10.0% 9.8% | | 0.894 | 0.004 |
|  |  | 1 2 | | 8183 | phenytoin |  | | 144 61 | 6.9% 3.8% | | <0.001 | 0.139 |
|  |  | 1 2 | | 623400 | lacosamide |  | | 143 128 | 6.9% 8.0% | | 0.212 | 0.041 |
|  |  | 1 2 | | 39998 | zonisamide |  | | 162 109 | 7.8% 6.8% | | 0.242 | 0.039 |
|  |  | 1 2 | | 2002 | carbamazepine |  | | 173 142 | 8.3% 8.8% | | 0.584 | 0.018 |
|  |  | 1 2 | | CN101 | OPIOID ANALGESICS |  | | 651 879 | 31.3% 54.6% | | <0.001 | 0.485 |
|  |  | 1 2 | | CN302 | BENZODIAZEPINE DERIVATIVE SEDATIVES/HYPNOTICS |  | | 1,015 902 | 48.8% 56.1% | | <0.001 | 0.146 |
|  |  | 1 2 | | CN309 | SEDATIVES/HYPNOTICS,OTHER |  | | 266 271 | 12.8% 16.8% | | 0.001 | 0.114 |
|  |  | 1 2 | | AM000 | ANTIMICROBIALS |  | | 948 1,131 | 45.6% 70.3% | | <0.001 | 0.517 |
|  |  | 1 2 | | AN000 | ANTINEOPLASTICS |  | | 127 169 | 6.1% 10.5% | | <0.001 | 0.160 |
|  |  | 1 2 | | 21241 | clobazam |  | | 78 78 | 3.8% 4.8% | | 0.100 | 0.054 |
|  |  | 1 2 | | 187832 | pregabalin |  | | 95 121 | 4.6% 7.5% | | <0.001 | 0.124 |
|  |  | 1 2 | | RE000 | RESPIRATORY TRACT MEDICATIONS |  | | 1,057 1,123 | 50.8% 69.8% | | <0.001 | 0.395 |
|  |  | 1 2 | | 4135 | ethosuximide |  | | 32 33 | 1.5% 2.1% | | 0.241 | 0.039 |
|  |  | 1 2 | | 1739745 | brivaracetam |  | | 27 40 | 1.3% 2.5% | | 0.007 | 0.087 |
|  |  | 1 2 | | 1356552 | perampanel |  | | 26 13 | 1.3% 0.8% | | 0.193 | 0.044 |
|  |  | 1 2 | | 8691 | primidone |  | | 21 16 | 1.0% 1.0% | | 0.963 | 0.002 |
|  |  | 1 2 | | 1482502 | eslicarbazepine |  | | 16 17 | 0.8% 1.1% | | 0.358 | 0.030 |
|  |  | 1 2 | | 2045371 | cannabidiol |  | | 11 10 | 0.5% 0.6% | | 0.711 | 0.012 |
|  |  | 1 2 | | 2265690 | cenobamate |  | | 10 19 | 0.5% 1.2% | | 0.017 | 0.077 |
|  |  | 1 2 | | CN200 | ANESTHETICS |  | | 348 572 | 16.7% 35.6% | | <0.001 | 0.438 |
|  |  | 1 2 | | CN550 | ANTIVERTIGO AGENTS |  | | 96 154 | 4.6% 9.6% | | <0.001 | 0.194 |
|  |  | 1 2 | | IM000 | IMMUNOLOGICAL AGENTS |  | | 489 775 | 23.5% 48.2% | | <0.001 | 0.532 |
|  |  | 1 2 | | AU000 | AUTONOMIC MEDICATIONS |  | | 551 572 | 26.5% 35.6% | | <0.001 | 0.197 |
|  | **Laboratory** | | | | | | | | | | | |
|  |  | Cohort | | |  | Mean ± SD | | Patients | % of Cohort | | P-Value | Std diff. |
|  |  | 1 2 | | 9083 | BMI | 33.3 +/- 8.6 36.1 +/- 8.0 | | 1,233 1,011 | 59.3% 62.8% | | <0.001 | 0.337 |
|  |  | 1 2 | |  | 0 - 0 kg/m2 |  | | 1,235 1,013 | 59.4% 63.0% | | 0.027 | 0.074 |
|  |  | 1 2 | | 9025 | Glucose [Mass/volume] in Serum, Plasma or Blood | 86.3 +/- 8.2 86.6 +/- 7.6 | | 625 898 | 30.0% 55.8% | | 0.574 | 0.029 |
|  |  | 1 2 | |  | 0 - 0 mg/dL |  | | 625 898 | 30.0% 55.8% | | <0.001 | 0.539 |
|  |  | 1 2 | | 9037 | Hemoglobin A1c/Hemoglobin.total in Blood | 5.1 +/- 0.4 5.2 +/- 0.3 | | 288 493 | 13.8% 30.6% | | 0.276 | 0.077 |
|  |  | 1 2 | |  | 0 - 0 % |  | | 288 493 | 13.8% 30.6% | | <0.001 | 0.412 |
| **Cohort 1 (N = 847) and cohort 2 (N = 847) characteristics after propensity score matching** | | | | | | | | | | | | |
|  | **Demographics** | | | | | | | | | | | |
|  |  | Cohort | | |  | Mean ± SD | | Patients | % of Cohort | | P-Value | Std diff. |
|  |  | 1 2 | | AI | Age at Index | 41.1 +/- 18.7 41.1 +/- 15.8 | | 847 847 | 100% 100% | | 0.980 | 0.001 |
|  |  | 1 2 | | 2106-3 | White |  | | 565 576 | 66.7% 68.0% | | 0.569 | 0.028 |
|  |  | 1 2 | | UNK | Unknown Race |  | | 152 139 | 17.9% 16.4% | | 0.402 | 0.041 |
|  |  | 1 2 | | F | Female |  | | 629 605 | 74.3% 71.4% | | 0.190 | 0.064 |
|  |  | 1 2 | | 2054-5 | Black or African American |  | | 84 83 | 9.9% 9.8% | | 0.935 | 0.004 |
|  |  | 1 2 | | M | Male |  | | 216 241 | 25.5% 28.5% | | 0.171 | 0.067 |
|  |  | 1 2 | | 2131-1 | Other Race |  | | 27 30 | 3.2% 3.5% | | 0.686 | 0.020 |
|  |  | 1 2 | | 2028-9 | Asian |  | | 12 13 | 1.4% 1.5% | | 0.840 | 0.010 |
|  | **Diagnosis** | | | | | | | | | | | |
|  |  | Cohort | | |  | Mean ± SD | | Patients | % of Cohort | | P-Value | Std diff. |
|  |  | 1 2 | | V00-Y99 | External causes of morbidity |  | | 141 140 | 16.6% 16.5% | | 0.948 | 0.003 |
|  |  | 1 2 | | F01-F99 | Mental, Behavioral and Neurodevelopmental disorders |  | | 507 497 | 59.9% 58.7% | | 0.621 | 0.024 |
|  |  | 1 2 | | E65-E68 | Overweight, obesity and other hyperalimentation |  | | 301 302 | 35.5% 35.7% | | 0.960 | 0.002 |
|  |  | 1 2 | | E28.2 | Polycystic ovarian syndrome |  | | 57 52 | 6.7% 6.1% | | 0.621 | 0.024 |
|  |  | 1 2 | | A00-B99 | Certain infectious and parasitic diseases |  | | 194 196 | 22.9% 23.1% | | 0.908 | 0.006 |
|  |  | 1 2 | | S00-S09 | Injuries to the head |  | | 97 92 | 11.5% 10.9% | | 0.700 | 0.019 |
|  |  | 1 2 | | I60-I69 | Cerebrovascular diseases |  | | 67 79 | 7.9% 9.3% | | 0.299 | 0.050 |
|  |  | 1 2 | | I10-I1A | Hypertensive diseases |  | | 174 174 | 20.5% 20.5% | | 1 | <0.001 |
|  |  | 1 2 | | J00-J99 | Diseases of the respiratory system |  | | 318 308 | 37.5% 36.4% | | 0.615 | 0.024 |
|  |  | 1 2 | | K00-K95 | Diseases of the digestive system |  | | 343 339 | 40.5% 40.0% | | 0.843 | 0.010 |
|  |  | 1 2 | | E70-E88 | Metabolic disorders |  | | 208 207 | 24.6% 24.4% | | 0.955 | 0.003 |
|  |  | 1 2 | | G40.9 | Epilepsy, unspecified |  | | 465 443 | 54.9% 52.3% | | 0.284 | 0.052 |
|  |  | 1 2 | | G40.2 | Localization-related (focal) (partial) symptomatic epilepsy and epileptic syndromes with complex partial seizures |  | | 123 119 | 14.5% 14.0% | | 0.781 | 0.013 |
|  |  | 1 2 | | G40.3 | Generalized idiopathic epilepsy and epileptic syndromes |  | | 128 121 | 15.1% 14.3% | | 0.631 | 0.023 |
|  |  | 1 2 | | G40.1 | Localization-related (focal) (partial) symptomatic epilepsy and epileptic syndromes with simple partial seizures |  | | 106 98 | 12.5% 11.6% | | 0.550 | 0.029 |
|  |  | 1 2 | | G40.4 | Other generalized epilepsy and epileptic syndromes |  | | 75 77 | 8.9% 9.1% | | 0.865 | 0.008 |
|  |  | 1 2 | | G40.8 | Other epilepsy and recurrent seizures |  | | 56 59 | 6.6% 7.0% | | 0.772 | 0.014 |
|  |  | 1 2 | | R56 | Convulsions, not elsewhere classified |  | | 503 521 | 59.4% 61.5% | | 0.371 | 0.043 |
|  |  | 1 2 | | G80-G83 | Cerebral palsy and other paralytic syndromes |  | | 45 47 | 5.3% 5.5% | | 0.830 | 0.010 |
|  |  | 1 2 | | G89-G99 | Other disorders of the nervous system |  | | 195 188 | 23.0% 22.2% | | 0.684 | 0.020 |
|  |  | 1 2 | | Z00-Z99 | Factors influencing health status and contact with health services |  | | 561 551 | 66.2% 65.1% | | 0.609 | 0.025 |
|  |  | 1 2 | | M00-M99 | Diseases of the musculoskeletal system and connective tissue |  | | 399 400 | 47.1% 47.2% | | 0.961 | 0.002 |
|  |  | 1 2 | | N00-N99 | Diseases of the genitourinary system |  | | 308 308 | 36.4% 36.4% | | 1 | <0.001 |
|  |  | 1 2 | | C00-D49 | Neoplasms |  | | 148 135 | 17.5% 15.9% | | 0.397 | 0.041 |
|  |  | 1 2 | | Q00-Q99 | Congenital malformations, deformations and chromosomal abnormalities |  | | 103 101 | 12.2% 11.9% | | 0.881 | 0.007 |
|  |  | 1 2 | | D50-D89 | Diseases of the blood and blood-forming organs and certain disorders involving the immune mechanism |  | | 104 111 | 12.3% 13.1% | | 0.609 | 0.025 |
|  |  | 1 2 | | E00-E07 | Disorders of thyroid gland |  | | 115 119 | 13.6% 14.0% | | 0.778 | 0.014 |
|  |  | 1 2 | | E50-E64 | Other nutritional deficiencies |  | | 98 114 | 11.6% 13.5% | | 0.240 | 0.057 |
|  |  | 1 2 | | S90-S99 | Injuries to the ankle and foot |  | | 55 58 | 6.5% 6.8% | | 0.770 | 0.014 |
|  |  | 1 2 | | S80-S89 | Injuries to the knee and lower leg |  | | 59 61 | 7.0% 7.2% | | 0.850 | 0.009 |
|  |  | 1 2 | | S60-S69 | Injuries to the wrist, hand and fingers |  | | 47 43 | 5.5% 5.1% | | 0.665 | 0.021 |
|  |  | 1 2 | | S40-S49 | Injuries to the shoulder and upper arm |  | | 35 33 | 4.1% 3.9% | | 0.804 | 0.012 |
|  |  | 1 2 | | T80-T88 | Complications of surgical and medical care, not elsewhere classified |  | | 38 40 | 4.5% 4.7% | | 0.817 | 0.011 |
|  |  | 1 2 | | S30-S39 | Injuries to the abdomen, lower back, lumbar spine, pelvis and external genitals |  | | 31 23 | 3.7% 2.7% | | 0.269 | 0.054 |
|  |  | 1 2 | | S10-S19 | Injuries to the neck |  | | 23 21 | 2.7% 2.5% | | 0.760 | 0.015 |
|  |  | 1 2 | | T66-T78 | Other and unspecified effects of external causes |  | | 42 44 | 5.0% 5.2% | | 0.825 | 0.011 |
|  |  | 1 2 | | T14-T14 | Injury of unspecified body region (T14) |  | | 30 26 | 3.5% 3.1% | | 0.587 | 0.026 |
|  |  | 1 2 | | I30-I5A | Other forms of heart disease |  | | 95 96 | 11.2% 11.3% | | 0.939 | 0.004 |
|  |  | 1 2 | | I80-I89 | Diseases of veins, lymphatic vessels and lymph nodes, not elsewhere classified |  | | 31 34 | 3.7% 4.0% | | 0.704 | 0.018 |
|  |  | 1 2 | | I70-I79 | Diseases of arteries, arterioles and capillaries |  | | 30 29 | 3.5% 3.4% | | 0.895 | 0.006 |
|  |  | 1 2 | | H60-H95 | Diseases of the ear and mastoid process |  | | 130 134 | 15.3% 15.8% | | 0.789 | 0.013 |
|  |  | 1 2 | | U00-U85 | Codes for special purposes |  | | 35 43 | 4.1% 5.1% | | 0.354 | 0.045 |
|  |  | 1 2 | | G47 | Sleep disorders |  | | 198 201 | 23.4% 23.7% | | 0.864 | 0.008 |
|  |  | 1 2 | | G43 | Migraine |  | | 162 170 | 19.1% 20.1% | | 0.624 | 0.024 |
|  |  | 1 2 | | G44 | Other headache syndromes |  | | 90 84 | 10.6% 9.9% | | 0.631 | 0.023 |
|  |  | 1 2 | | R51 | Headache |  | | 173 168 | 20.4% 19.8% | | 0.762 | 0.015 |
|  |  | 1 2 | | R55 | Syncope and collapse |  | | 80 74 | 9.4% 8.7% | | 0.612 | 0.025 |
|  |  | 1 2 | | R53 | Malaise and fatigue |  | | 99 95 | 11.7% 11.2% | | 0.760 | 0.015 |
|  |  | 1 2 | | R00-R09 | Symptoms and signs involving the circulatory and respiratory systems |  | | 278 295 | 32.8% 34.8% | | 0.383 | 0.042 |
|  |  | 1 2 | | R25-R29 | Symptoms and signs involving the nervous and musculoskeletal systems |  | | 123 132 | 14.5% 15.6% | | 0.541 | 0.030 |
|  |  | 1 2 | | R10-R19 | Symptoms and signs involving the digestive system and abdomen |  | | 254 255 | 30.0% 30.1% | | 0.958 | 0.003 |
|  | **Procedure** | | | | | | | | | | | |
|  |  | Cohort | | |  | Mean ± SD | | Patients | % of Cohort | | P-Value | Std diff. |
|  |  | 1 2 | | 1010251 | Radiology Procedures |  | | 424 411 | 50.1% 48.5% | | 0.528 | 0.031 |
|  |  | 1 2 | | 1011136 | Pathology and Laboratory Procedures |  | | 378 375 | 44.6% 44.3% | | 0.883 | 0.007 |
|  |  | 1 2 | | 1012569 | Medicine Services and Procedures |  | | 469 446 | 55.4% 52.7% | | 0.262 | 0.055 |
|  |  | 1 2 | | 1013625 | Evaluation and Management |  | | 475 468 | 56.1% 55.3% | | 0.732 | 0.017 |
|  |  | 1 2 | | 1003143 | Surgery |  | | 351 343 | 41.4% 40.5% | | 0.693 | 0.019 |
|  | **Medication** | | | | | | | | | | | |
|  |  | Cohort | | |  | Mean ± SD | | Patients | % of Cohort | | P-Value | Std diff. |
|  |  | 1 2 | | CN600 | ANTIDEPRESSANTS |  | | 490 471 | 57.9% 55.6% | | 0.351 | 0.045 |
|  |  | 1 2 | | CN300 | SEDATIVES/HYPONTICS |  | | 498 482 | 58.8% 56.9% | | 0.431 | 0.038 |
|  |  | 1 2 | | CN700 | ANTIPSYCHOTICS |  | | 208 204 | 24.6% 24.1% | | 0.821 | 0.011 |
|  |  | 1 2 | | CN800 | CNS STIMULANTS |  | | 146 159 | 17.2% 18.8% | | 0.411 | 0.040 |
|  |  | 1 2 | | CV000 | CARDIOVASCULAR MEDICATIONS |  | | 579 588 | 68.4% 69.4% | | 0.637 | 0.023 |
|  |  | 1 2 | | 114477 | levetiracetam |  | | 343 339 | 40.5% 40.0% | | 0.843 | 0.010 |
|  |  | 1 2 | | 28439 | lamotrigine |  | | 266 259 | 31.4% 30.6% | | 0.713 | 0.018 |
|  |  | 1 2 | | 38404 | topiramate |  | | 217 233 | 25.6% 27.5% | | 0.379 | 0.043 |
|  |  | 1 2 | | 40254 | valproate |  | | 133 130 | 15.7% 15.3% | | 0.840 | 0.010 |
|  |  | 1 2 | | 25480 | gabapentin |  | | 158 150 | 18.7% 17.7% | | 0.614 | 0.024 |
|  |  | 1 2 | | 32624 | oxcarbazepine |  | | 83 75 | 9.8% 8.9% | | 0.504 | 0.032 |
|  |  | 1 2 | | 8183 | phenytoin |  | | 33 36 | 3.9% 4.3% | | 0.712 | 0.018 |
|  |  | 1 2 | | 623400 | lacosamide |  | | 71 69 | 8.4% 8.1% | | 0.860 | 0.009 |
|  |  | 1 2 | | 39998 | zonisamide |  | | 64 56 | 7.6% 6.6% | | 0.449 | 0.037 |
|  |  | 1 2 | | 2002 | carbamazepine |  | | 77 73 | 9.1% 8.6% | | 0.732 | 0.017 |
|  |  | 1 2 | | CN101 | OPIOID ANALGESICS |  | | 397 386 | 46.9% 45.6% | | 0.592 | 0.026 |
|  |  | 1 2 | | CN302 | BENZODIAZEPINE DERIVATIVE SEDATIVES/HYPNOTICS |  | | 453 444 | 53.5% 52.4% | | 0.661 | 0.021 |
|  |  | 1 2 | | CN309 | SEDATIVES/HYPNOTICS,OTHER |  | | 139 134 | 16.4% 15.8% | | 0.741 | 0.016 |
|  |  | 1 2 | | AM000 | ANTIMICROBIALS |  | | 527 523 | 62.2% 61.7% | | 0.841 | 0.010 |
|  |  | 1 2 | | AN000 | ANTINEOPLASTICS |  | | 78 79 | 9.2% 9.3% | | 0.933 | 0.004 |
|  |  | 1 2 | | 21241 | clobazam |  | | 37 43 | 4.4% 5.1% | | 0.492 | 0.033 |
|  |  | 1 2 | | 187832 | pregabalin |  | | 60 54 | 7.1% 6.4% | | 0.561 | 0.028 |
|  |  | 1 2 | | RE000 | RESPIRATORY TRACT MEDICATIONS |  | | 534 529 | 63.0% 62.5% | | 0.802 | 0.012 |
|  |  | 1 2 | | 4135 | ethosuximide |  | | 17 14 | 2.0% 1.7% | | 0.587 | 0.026 |
|  |  | 1 2 | | 1739745 | brivaracetam |  | | 14 13 | 1.7% 1.5% | | 0.846 | 0.009 |
|  |  | 1 2 | | 1356552 | perampanel |  | | 10 10 | 1.2% 1.2% | | 1 | <0.001 |
|  |  | 1 2 | | 8691 | primidone |  | | 10 10 | 1.2% 1.2% | | 1 | <0.001 |
|  |  | 1 2 | | 1482502 | eslicarbazepine |  | | 10 10 | 1.2% 1.2% | | 1 | <0.001 |
|  |  | 1 2 | | 2045371 | cannabidiol |  | | 10 10 | 1.2% 1.2% | | 1 | <0.001 |
|  |  | 1 2 | | 2265690 | cenobamate |  | | 10 10 | 1.2% 1.2% | | 1 | <0.001 |
|  |  | 1 2 | | CN200 | ANESTHETICS |  | | 215 211 | 25.4% 24.9% | | 0.823 | 0.011 |
|  |  | 1 2 | | CN550 | ANTIVERTIGO AGENTS |  | | 64 53 | 7.6% 6.3% | | 0.292 | 0.051 |
|  |  | 1 2 | | IM000 | IMMUNOLOGICAL AGENTS |  | | 320 315 | 37.8% 37.2% | | 0.802 | 0.012 |
|  |  | 1 2 | | AU000 | AUTONOMIC MEDICATIONS |  | | 256 241 | 30.2% 28.5% | | 0.423 | 0.039 |
|  | **Laboratory** | | | | | | | | | | | |
|  |  | Cohort | | |  | Mean ± SD | | Patients | % of Cohort | | P-Value | Std diff. |
|  |  | 1 2 | | 9083 | BMI | 34.2 +/- 9.1 35.1 +/- 8.1 | | 496 505 | 58.6% 59.6% | | 0.134 | 0.095 |
|  |  | 1 2 | |  | 0 - 0 kg/m2 |  | | 496 506 | 58.6% 59.7% | | 0.621 | 0.024 |
|  |  | 1 2 | | 9025 | Glucose [Mass/volume] in Serum, Plasma or Blood | 86.4 +/- 8.3 86.2 +/- 7.8 | | 361 349 | 42.6% 41.2% | | 0.744 | 0.024 |
|  |  | 1 2 | |  | 0 - 0 mg/dL |  | | 361 349 | 42.6% 41.2% | | 0.555 | 0.029 |
|  |  | 1 2 | | 9037 | Hemoglobin A1c/Hemoglobin.total in Blood | 5.1 +/- 0.5 5.2 +/- 0.3 | | 176 173 | 20.8% 20.4% | | 0.721 | 0.038 |
|  |  | 1 2 | |  | 0 - 0 % |  | | 176 173 | 20.8% 20.4% | | 0.857 | 0.009 |

# **Supplement 5:** Illustrative sample size calculation for a future trial

**Objective:** To provide an illustrative sample size estimate for a future randomised trial evaluating metformin versus licensed weight-management comparators in people with epilepsy without evidence of diabetes, using the net potentially seizure-related morbidity outcome as the primary endpoint.

**Effect estimate:** We used the hazard ratio observed in this study for the net potentially seizure-related morbidity outcome (HR = 0.729).

**Assumptions:** Two-sided alpha = 0.05, power = 80%, and 1:1 allocation between trial arms, with analysis by log-rank test / Cox proportional hazards model.

**Required number of events:** Using the Schoenfeld approximation for time-to-event analyses, the required number of events (E) is estimated as:

E = (Z(1−α/2) + Z(1−β))² / ((ln(HR))² × p × (1 − p))

where p = 0.5 for equal allocation, Z(1−α/2) = 1.96, and Z(1−β) = 0.84.

Substituting HR = 0.729:

ln(HR) = ln(0.729) ≈ −0.316

Therefore:

E = (1.96 + 0.84)² / ((0.316)² × 0.25) ≈ 7.84 / 0.025 ≈ 315 events

**Translation to total sample size:** The Kaplan–Meier survival probability at 10 years for the net potentially seizure-related morbidity outcome was 28.28% in the metformin group and 12.44% in the licensed weight-management comparator group. The corresponding 10-year cumulative event risks were therefore 71.72% and 87.56%, respectively. Taking the mean event risk across arms (~79.6%), the total sample size required to observe ~315 events is estimated as:

N ≈ 315 / 0.796 ≈ 396 participants

**Summary estimate:** This corresponds to approximately 400 participants in total (about 200 per arm) followed for 10 years, noting that larger numbers would be required in practice to account for attrition, non-adherence, and incomplete follow-up.

**Reference**

- Schoenfeld DA. Sample-size formula for the proportional-hazards regression model. *Biometrics* 1983;39(2):499–503.
